# Supplementary material for: Centriolar Satellites Control GABARAP Ubiquitination and GABARAP-Mediated Autophagy
Source: Curr Biol. 2017 Jul 24;27(14):2123–2136.e7. doi: 10.1016/j.cub.2017.06.021 (PMC5526835; doi:10.1016/j.cub.2017.06.021)
Supplement: Document S1. Figures S1–S5 [file mmc1.pdf]

Current Biology, Volume *27*

Supplemental Information

Centriolar Satellites Control GABARAP Ubiquitination and GABARAP-Mediated Autophagy

Justin Joachim, Minoo Razi, Delphine Judith, Martina Wirth, Emily Calamita, Vesela Encheva, Brian D. Dynlacht, Ambrosius P. Snijders, Nicola O'Reilly, Harold B.J. Jefferies, and Sharon A. Tooze

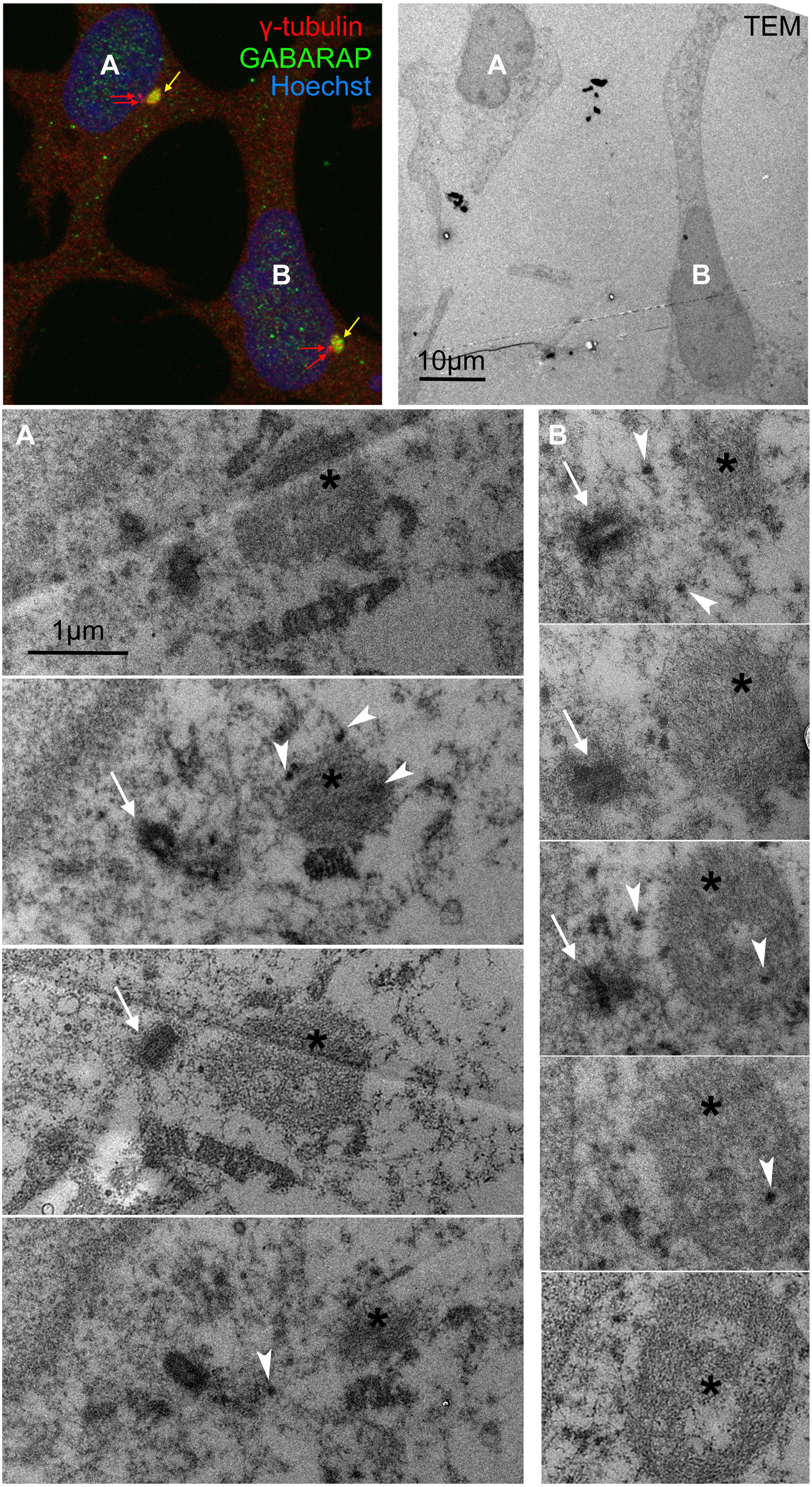

γ-tubulin
GABARAP
Hoechst
A
B
TEM
A
B
10µm
A
1µm
B

Figure S1. Correlative light and electron microscopy of centrosomal GABARAP. See also Figure 2.

HEK293A cells (**A** and **B**) fixed and Triton X-100 permeabilized were labelled with the indicated antibodies for confocal microscopy followed by transmission electron microscopy (TEM). Red arrows, γ-tubulin positive GABARAP negative centrioles. Yellow arrows, γ-tubulin positive and GABARAP positive pericentriolar material. TEM sections from the same cell (A or B) are shown. *, pericentriolar material; white arrows, centrioles; white arrowheads, detergent-resistant electron-dense granules, likely centriolar satellites.

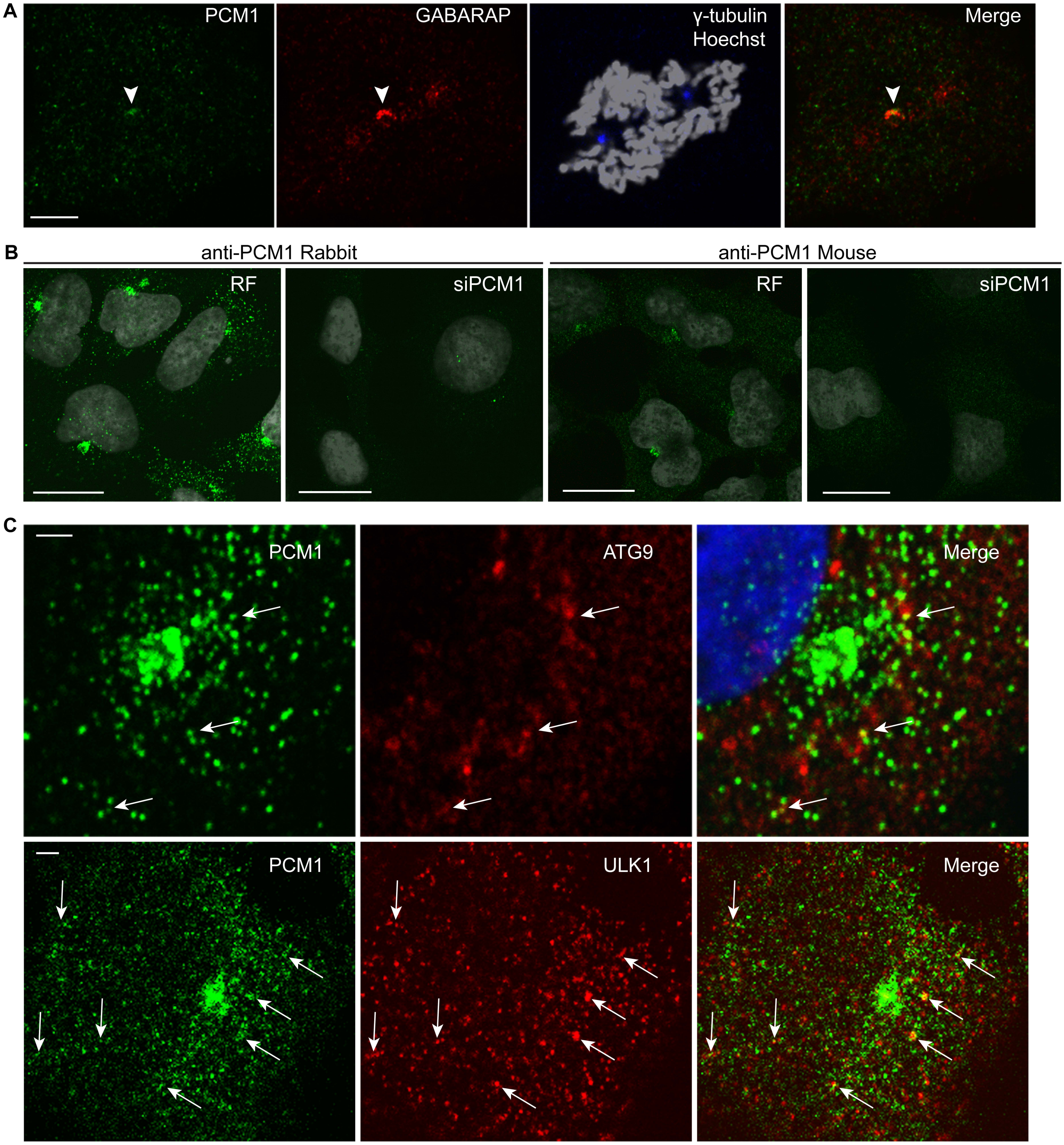
A
PCM1
GABARAP
γ-tubulin
Hoechst
Merge
B
anti-PCM1 Rabbit
anti-PCM1 Mouse
RF
siPCM1
RF
siPCM1
C
PCM1
ATG9
Merge
PCM1
ULK1
Merge

Figure S2. Validation of anti-PCM1 staining and PCM1 colocalization with autophagy markers. See also Figures 2 and 3.

A HEK293A cells incubated in full medium were fixed and labelled with the indicated antibodies. Scale bar, 5 μm. Mitotic cell is shown. Mouse anti-PCM1, rabbit anti-GABARAP and goat anti-γ tubulin antibodies were used. **B** HEK293A cells were treated with RF or PCM1 siRNA for 72 hr then, fixed, and labelled with the indicated PCM1 antibodies. Scale bar, 20 μm. **C** HEK293A cells starved for 2 hr in EBSS (ULK1 staining only) then fixed and labelled with the indicated antibodies. Scale bars, 2 μm. ATG9 labelled cell was incubated in full medium. Arrows indicate colocalization between PCM1 and autophagy markers. Hoechst DNA staining is shown in blue in the merge (ATG9 only). Rabbit anti-PCM1 was used with hamster anti-ATG9 antibody; mouse anti-PCM1 was used with rabbit anti-ULK1 antibody.

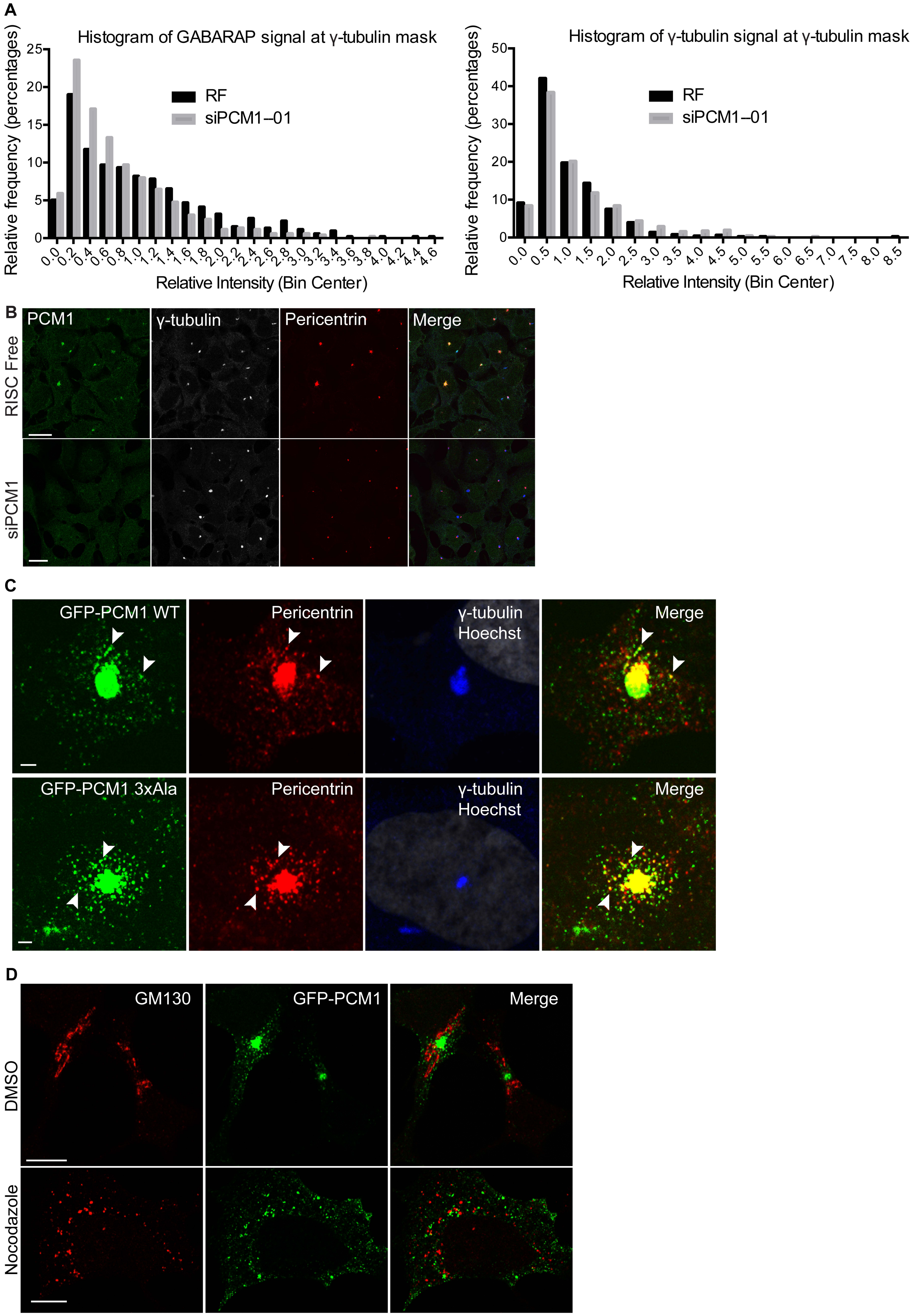
A
Histogram of GABARAP signal at γ-tubulin mask
Relative frequency (percentages)
25
20
15
10
5
0
RF
siPCM1–01
0.0 0.2 0.4 0.6 0.8 1.0 1.2 1.4 1.6 1.8 2.0 2.2 2.4 2.6 2.8 3.0 3.2 3.4 3.6 3.8 4.0 4.2 4.4 4.6
Relative Intensity (Bin Center)
Histogram of γ-tubulin signal at γ-tubulin mask
Relative frequency (percentages)
50
40
30
20
10
0
RF
siPCM1–01
0.0 0.5 1.0 1.5 2.0 2.5 3.0 3.5 4.0 4.5 5.0 5.5 6.0 6.5 7.0 7.5 8.0 8.5
Relative Intensity (Bin Center)
B
PCM1
γ-tubulin
Pericentrin
Merge
RISC Free
siPCM1
C
GFP-PCM1 WT
Pericentrin
γ-tubulin
Hoechst
Merge
GFP-PCM1 3xAla
Pericentrin
γ-tubulin
Hoechst
Merge
D
GM130
GFP-PCM1
Merge
DMSO
Nocodazole

Figure S3. PCM1 depletion affects centrosomal GABARAP and Pericentrin localization and the PCM1 LIR does not regulate colocalization with Pericentrin. See also Figure 4.

A Frequency distribution of data shown in Figure 4B. Data is normalized to the mean intensity of RF signals and assigned to the nearest bin center. **B** HEK293A cells were treated with RF or PCM1 siRNA for 72 hr then, fixed, and labelled with the indicated antibodies. Scale bar, 20 µm. Mouse anti-PCM1, goat anti-γ tubulin, and rabbit anti-Pericentrin antibodies were used. **C** HEK293A cells expressing GFP-PCM1 wild-type or 3xAla LIR mutant starved for 2 hr in EBSS then fixed and labelled with rabbit anti-Pericentrin and mouse anti-γ tubulin. Scale bars, 2µm. Hoechst DNA staining is shown in the γ-tubulin channel and false-coloured white. Merge shows red and green channels. Arrowheads, GFP-PCM1 WT or 3xAla colocalization with Pericentrin puncta. **D** HEK293A cells expressing GFP-PCM1 were treated with 50 µM nocodazole for 5 hr in total prior to fixation and labelled with mouse anti-GM130 antibodies. Scale bar, 10 µm.

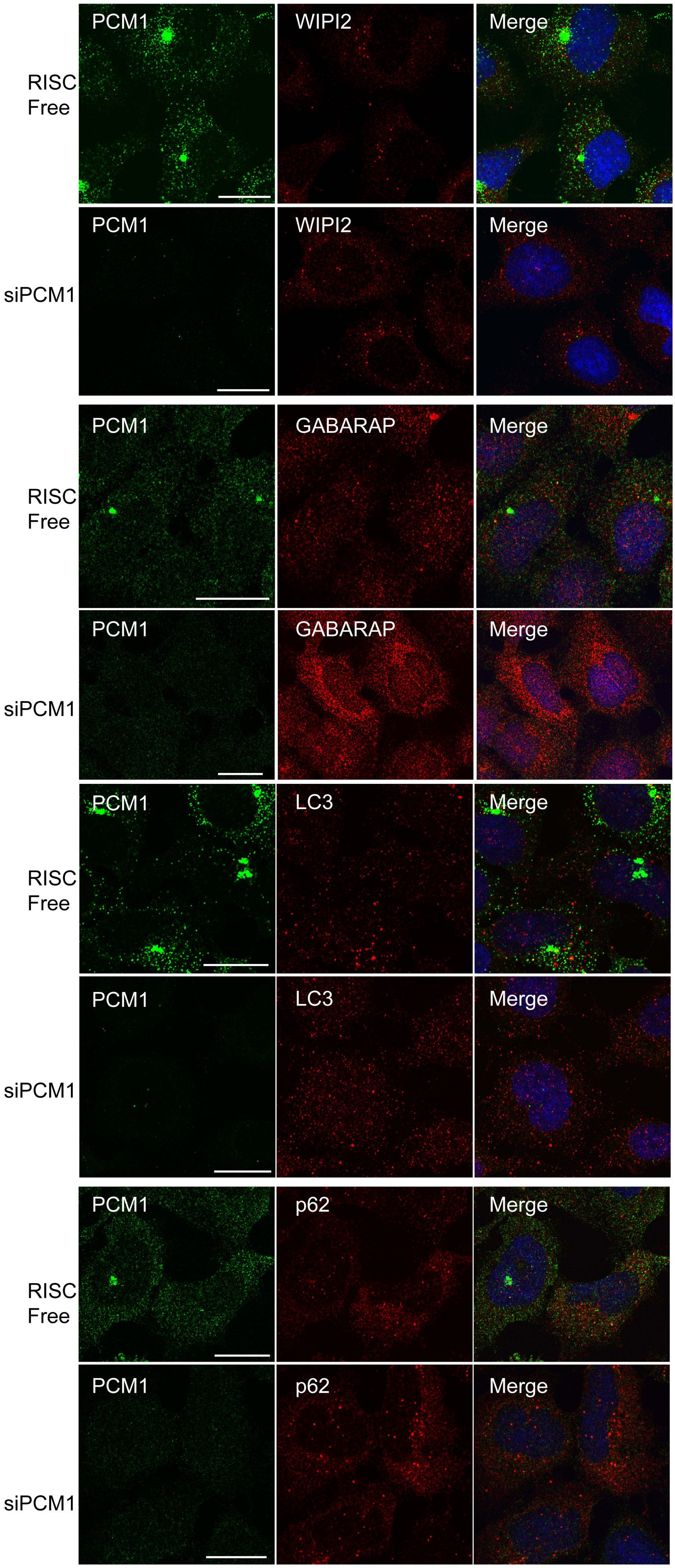
RISC Free
siPCM1
RISC Free
siPCM1
RISC Free
siPCM1
RISC Free
siPCM1
PCM1
WIPI2
GABARAP
LC3
p62
Merge

Figure S4. Depletion of PCM1 increases GABARAP but not LC3B specific autophagosome formation. See also Figure 4.

HEK293A cells were treated with RF or PCM1 siRNA for 72 hr and incubated in EBSS for 2 hr, fixed, and labelled with the indicated antibodies. Scale bars, 20 μm. Mouse anti-WIPI2, rabbit anti-PCM1, rabbit anti-GABARAP, mouse anti-LC3B, guinea pig anti-p62. For co-staining with rabbit anti-GABARAP, mouse anti-PCM1 was used.

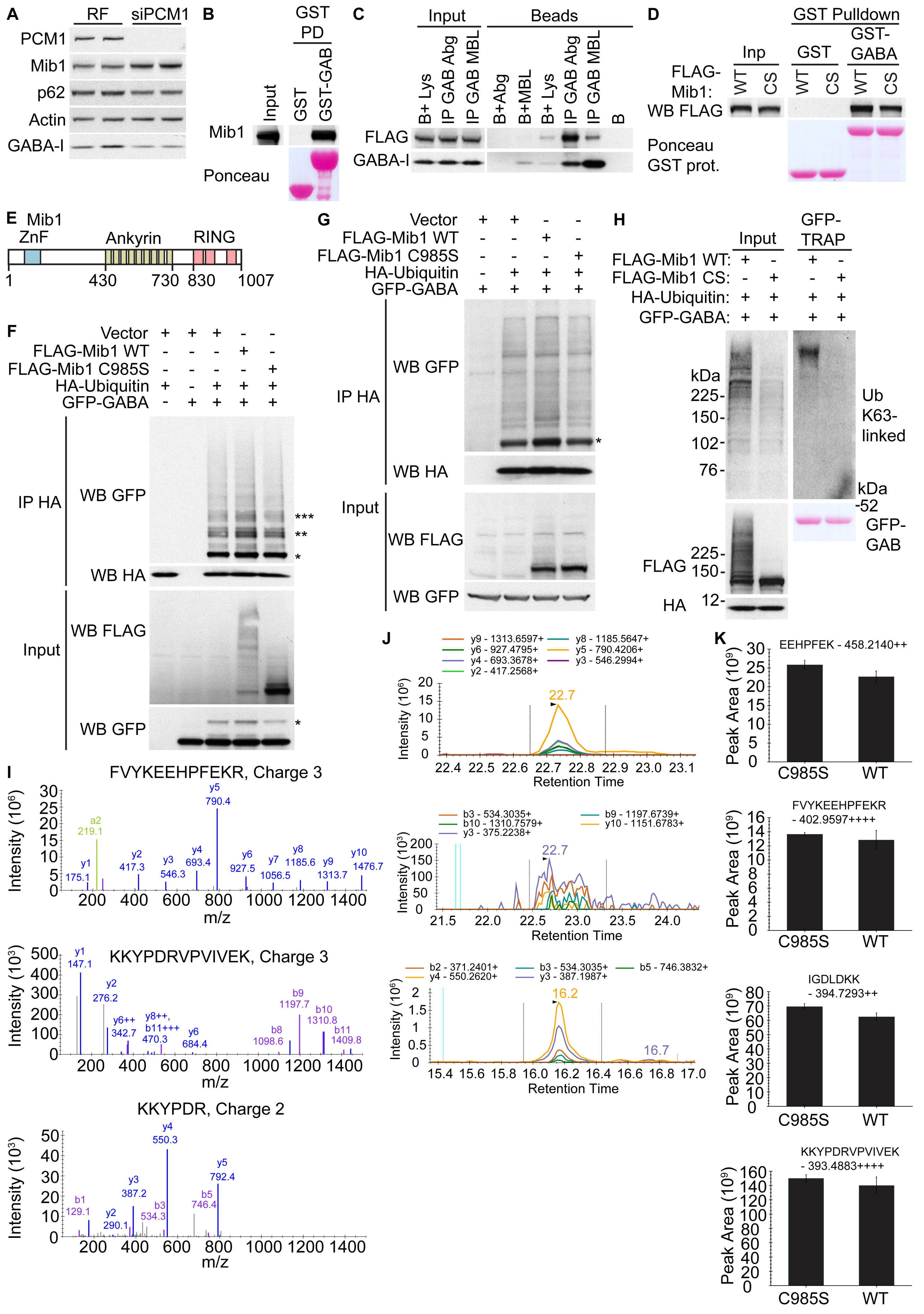

A
RF
siPCM1
PCM1
Mib1
p62
Actin
GABA-I
B
GST PD
Input
GST
GST-GAB
Mib1
Ponceau
C
Input
Beads
B+ Lys
IP GAB Abg
IP GAB MBL
B+Abg
B+MBL
B+ Lys
IP GAB Abg
IP GAB MBL
B
FLAG
GABA-I
D
GST Pulldown
Inp
GST
GST-GABA
FLAG-Mib1:
WT
CS
WB FLAG
Ponceau
GST prot.
E
Mib1
ZnF
Ankyrin
RING
1
430
730
830
1007
F
Vector
FLAG-Mib1 WT
FLAG-Mib1 C985S
HA-Ubiquitin
GFP-GABA
IP HA
WB GFP
***
**
*
WB HA
Input
WB FLAG
WB GFP
G
Vector
FLAG-Mib1 WT
FLAG-Mib1 C985S
HA-Ubiquitin
GFP-GABA
IP HA
WB GFP
WB HA
Input
WB FLAG
WB GFP
H
Input
GFP-TRAP
FLAG-Mib1 WT:
FLAG-Mib1 CS:
HA-Ubiquitin:
GFP-GABA:
kDa
225-
150-
102-
76-
Ub K63-linked
kDa
-52
GFP-GAB
FLAG
225-
150-
HA
12-
I
FVYKEEHPFEKR, Charge 3
Intensity (10⁶)
a2 219.1
y1 175.1
y2 417.3
y3 546.3
y4 693.4
y5 790.4
y6 927.5
y7 1056.5
y8 1185.6
y9 1313.7
y10 1476.7
m/z
KKYPDRVPVIVEK, Charge 3
Intensity (10³)
y1 147.1
y2 276.2
y6++ 342.7
y8++, b11+++ 470.3
y6 684.4
b8 1098.6
b9 1197.7
b10 1310.8
b11 1409.8
m/z
KKYPDR, Charge 2
Intensity (10³)
b1 129.1
y2 290.1
y3 387.2
b3 534.3
y4 550.3
b5 746.4
y5 792.4
m/z
J
y9 - 1313.6597+
y8 - 1185.5647+
y6 - 927.4795+
y5 - 790.4206+
y4 - 693.3678+
y3 - 546.2994+
y2 - 417.2568+
Intensity (10⁶)
22.7
Retention Time
b3 - 534.3035+
b9 - 1197.6739+
b10 - 1310.7579+
y10 - 1151.6783+
y3 - 375.2238+
Intensity (10³)
22.7
Retention Time
b2 - 371.2401+
b3 - 534.3035+
b5 - 746.3832+
y4 - 550.2620+
y3 - 387.1987+
Intensity (10⁶)
16.2
16.7
Retention Time
K
EEHPFEK - 458.2140++
Peak Area (10⁹)
C985S
WT
FVYKEEHPFEKR - 402.9597++++
Peak Area (10⁹)
C985S
WT
IGDLDKK - 394.7293++
Peak Area (10⁹)
C985S
WT
KKYPDRVPVIVEK - 393.4883++++
Peak Area (10⁹)
C985S
WT

Figure S5. Mib1 promotes ubiquitination of GABARAP. See also Figure 7.

A HEK293A cells were treated with RF or PCM1 siRNA for 72 hr then immunoblotted. **B** HEK293A cells were incubated with recombinant GST or GST-GABARAP beads and immunoblotted. PD, pulldown. **C** Anti-GABARAP immunoprecipitate from HEK293A cells analyzed by immunoblotting. Abg, anti-GABARAP from Abgent. MBL, anti-GABARAP from MBL. Lys, HEK293A lysate. B, beads. **D** HEK293A cells expressing the indicated constructs were incubated with recombinant GST or GST-GABARAP beads and immunoblotted. Prot, protein; WT, wild-type; CS, C997S mutant. **E** Primary structure of human Mib1 showing domains. **F** Immunoprecipitation of U2OS cells lysed in boiling SDS buffer and expressing the indicated constructs and immunoblot. *, **, ***, mono, di and tri ubiquitinated GFP-GABARAP, respectively. WT, wild-type. **G** Immunoprecipitation of HEK293A cells lysed in TNTE buffer without N-Ethylmaleimide and expressing the indicated constructs and immunoblot. *, monoubiquitinated GFP-GABARAP. WT, wild-type. **H** GFP-TRAP of HEK293A cells expressing the indicated constructs and immunoblot. Immunoprecipitates were stringently washed in denaturing buffer. GAB, GABARAP; WT, wild-type; CS, C985S mutant. **I** Identification of GABARAP ubiquitination sites by LC MS/MS. GABARAP ubiquitination sites were identified by mass spectrometry following protein digestion with trypsin and LC MS/MS analysis using the QExactive MS. Two ubiquitination sites were detected including lysine 13 (K13) and lysine 23 (K23). The sites were identified with MaxQuant where the FDR was restricted to 1 % on peptide and site level. Top, MS2 fragmentation spectra of the FVYKEEHPFEK(diGly)R peptide containing K13. All matching b and y ions are annotated with their measured m/z (mass to charge). Middle and bottom, MS2 fragmentation spectra of peptides K(diGly)KYPDRVPVIVEK and K(diGly)KYPDR containing K23 ubiquitination site. The two peptides are a result of a missed cleavage and both contain the same ubiquitinated lysine. All matching y and b ions are annotated with their measured m/z (mass to charge). **J** Detection of GABARAP ubiquitination peptides using PRM. Three peptides of the GABARAP protein containing ubiquitinated lysines were detected using LC MS/MS. To further confirm their presence/absence a PRM-based targeted assay was developed using Skyline software. The observed MS2 signals (transitions) were integrated with Skyline and manually verified. Top, Co-eluting transitions of the K13 containing peptide FVYKEEHPFEK(diGly)R. The co-elution profiles of seven detectable y ions are annotated with their measured m/z (mass to charge) values. Middle and bottom, the K23 ubiquitination site was detected on two different peptides. Peptide K(diGly)KYPDRVPVIVEK (middle) and peptide K(diGly)KYPDR (bottom). For both peptides the co-elution profiles of five b and y ions are annotated with their measured m/z (mass to charge) values. **K** Quantitation of normalization peptides. To check for differences in the total amount of the GABARAP protein recovery, four unmodified peptides were also quantified. The peak areas of these normalization peptides were obtained using MS1 integration in Skyline. Each peak area was measured in three technical replicates. The mean peak area of the three measurements is displayed and the error bars indicate standard errors. Peak area calculations of the normalization peptides did not reveal any major differences in GABARAP loading between the different conditions. (Top) Peak areas of peptide EEHPFEK, (middle-top) peak areas of peptide FVYKEEHPFEKR, (middle-bottom) peak areas of peptide IGDLDKK, (bottom) peak areas of peptide KKYPDRVPVIVEK.
