# Supplementary material for: Centriolar Satellites Control GABARAP Ubiquitination and GABARAP-Mediated Autophagy
Source: Curr Biol. 2017 Jul 24;27(14):2123–2136.e7. doi: 10.1016/j.cub.2017.06.021 (PMC5526835; doi:10.1016/j.cub.2017.06.021)
Supplement: Document S2. Article plus Supplemental Information [file mmc2.pdf]

Article

Current Biology

Centriolar Satellites Control GABARAP Ubiquitination and GABARAP-Mediated Autophagy

Graphical Abstract

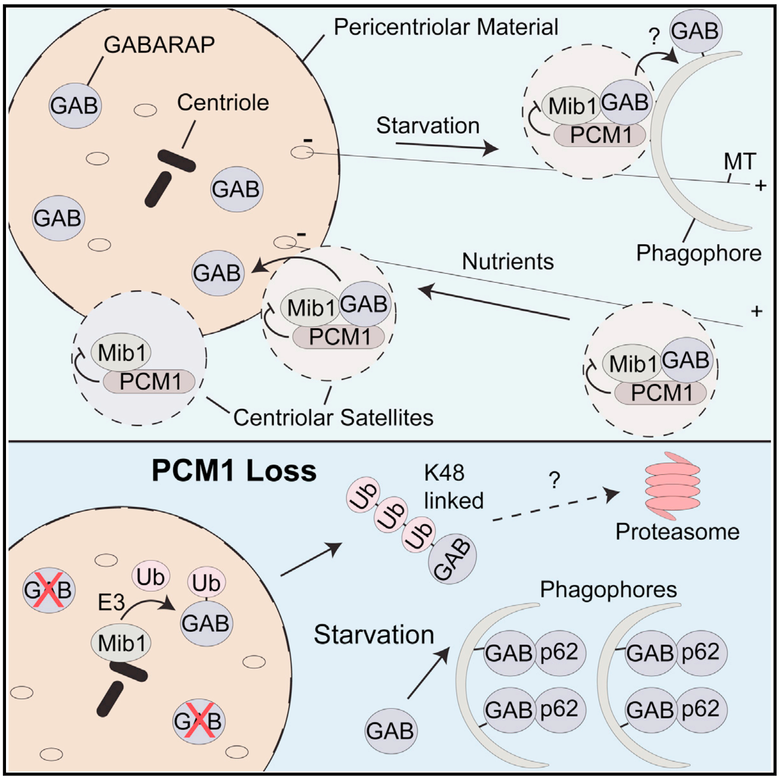

Authors

Justin Joachim, Minoo Razi, Delphine Judith, ..., Nicola O'Reilly, Harold B.J. Jefferies, Sharon A. Tooze

Correspondence

sharon.tooze@crick.ac.uk

In Brief

Joachim et al. show PCM1-positive centriolar satellites regulate the formation of GABARAP-positive autophagosomes. GABARAP stability is regulated by PCM1, most likely through Mib1-driven ubiquitination. This study reveals new insights into the poorly understood communication between the centrosome and autophagosomes during starvation-induced autophagy.

Highlights

- GABARAP binds directly to the centriolar satellite protein PCM1 through a LIR motif
- GABARAP-PCM1-positive centriolar satellites are found at early-stage autophagosomes
- PCM1 regulates GABARAP-specific autophagosome formation and GABARAP degradation
- The centriolar satellite E3 ligase Mib1 drives ubiquitination of GABARAP

Joachim et al., 2017, Current Biology *27*, 2123–2136
July 24, 2017 © 2017 The Author(s). Published by Elsevier Ltd.
http://dx.doi.org/10.1016/j.cub.2017.06.021

Current Biology
Article

Centriolar Satellites Control GABARAP Ubiquitination and GABARAP-Mediated Autophagy

Justin Joachim,[1] Minoo Razi,[1] Delphine Judith,[1] Martina Wirth,[1] Emily Calamita,[1] Vesela Encheva,[2] Brian D. Dynlacht,[4] Ambrosius P. Snijders,[2] Nicola O'Reilly,[3] Harold B.J. Jefferies,[1] and Sharon A. Tooze[1,5,*]
[1]Molecular Cell Biology of Autophagy
[2]Mass Spectrometry
[3]Peptide Chemistry
The Francis Crick Institute, 1 Midland Road, London NW1 1AT, UK
[4]Department of Pathology and NYU Cancer Institute, New York University School of Medicine, Smilow Research Building, 522 First Avenue, New York, NY 10016, USA
[5]Lead Contact
*Correspondence: sharon.tooze@crick.ac.uk
http://dx.doi.org/10.1016/j.cub.2017.06.021

SUMMARY

Autophagy maintains cellular health and homeostasis during stress by delivering cytosolic material captured by autophagosomes to lysosomes for degradation. Autophagosome formation is complex: initiated by the recruitment of autophagy (Atg) proteins to the formation site, it is sustained by activation of Atg proteins to allow growth and closure of the autophagosome. How Atg proteins are translocated to the forming autophagosome is not fully understood. Transport of the ATG8 family member GABARAP from the centrosome occurs during starvation-induced autophagosome biogenesis, but how centrosomal proteins regulate GABARAP localization is unknown. We show that the centriolar satellite protein PCM1 regulates the recruitment of GABARAP to the pericentriolar material. In addition to residing on the pericentriolar material, GABARAP marks a subtype of PCM1-positive centriolar satellites. GABARAP, but not another ATG8 family member LC3B, binds directly to PCM1 through a canonical LIR motif. Loss of PCM1 results in destabilization of GABARAP, but not LC3B, through proteasomal degradation. GABARAP instability is mediated through the centriolar satellite E3 ligase Mib1, which interacts with GABARAP through its substrate-binding region and promotes K48-linked ubiquitination of GABARAP. Ubiquitination of GABARAP occurs in the N terminus, a domain associated with ATG8-family-specific functions during autophagosome formation, on residues absent in the LC3 family. Furthermore, PCM1-GABARAP-positive centriolar satellites colocalize with forming autophagosomes. PCM1 enhances GABARAP/WIPI2/p62-positive autophagosome formation and flux but has no significant effect on LC3B-positive autophagosome formation. These data suggest a mechanism for how centriolar satellites can specifically regulate an ATG8 ortholog, the centrosomal GABARAP reservoir, and centrosome-autophagosome crosstalk.

INTRODUCTION

Autophagy is an intracellular recycling process that maintains cell homeostasis during stress. Autophagy occurs constitutively as a housekeeping process but is acutely upregulated upon insults, such as nutrient starvation. During autophagy, new vesicular organelles form, called autophagosomes. Autophagosome formation involves growth of a cup-shaped phagophore membrane that expands and encapsulates cargo, such as proteins and whole organelles [1]. These cargoes are trapped inside the closed, fully formed autophagosome. The autophagosome terminally fuses with the lysosome, resulting in destruction of the autophagosomal contents and recycling of macromolecules. Autophagy is an essential process for animal life and conserved from yeast to humans. The importance of autophagy for physiology is underlined by its involvement in pathologies, such as cancer, neurodegeneration, and infection.

Autophagosome formation is controlled by conserved signaling and machinery proteins called Atg proteins in yeast and mammals. These proteins localize to, and are markers of, the forming autophagosome. In mammals, formation is initiated by the ULK protein kinase complex, which phosphorylates and activates the ATG14-Beclin1-phosphatidylinositol 3-phosphate (PI3P) kinase complex. This results in a pool of PI3P at autophagosome formation sites on the endoplasmic reticulum, called omegasomes [2], and recruitment of DFCP1 and WIPI proteins, PI3P-binding effectors. WIPI2b recruits the ATG12–5-16L1 complex to the phagophore membrane, which mediates the lipidation of cytosolic ATG8 proteins by the lipid phosphatidylethanolamine and membrane association [3]. Vesicles containing the transmembrane protein ATG9 are also thought to contribute to autophagosome formation [4, 5]. In yeast, there is one ATG8 protein, but in mammals, there are multiple ATG8 orthologs. In humans, the ATG8 proteins comprise two subfamilies: LC3s and GABARAPs [6]. These ATG8 proteins function in formation and closure of the phagophore membrane and fusion of autophagosomes with lysosomes [7–9]. ATG8 proteins also bind autophagy receptor proteins, such as p62, which specifically

This is an open access article under the CC BY license (http://creativecommons.org/licenses/by/4.0/).

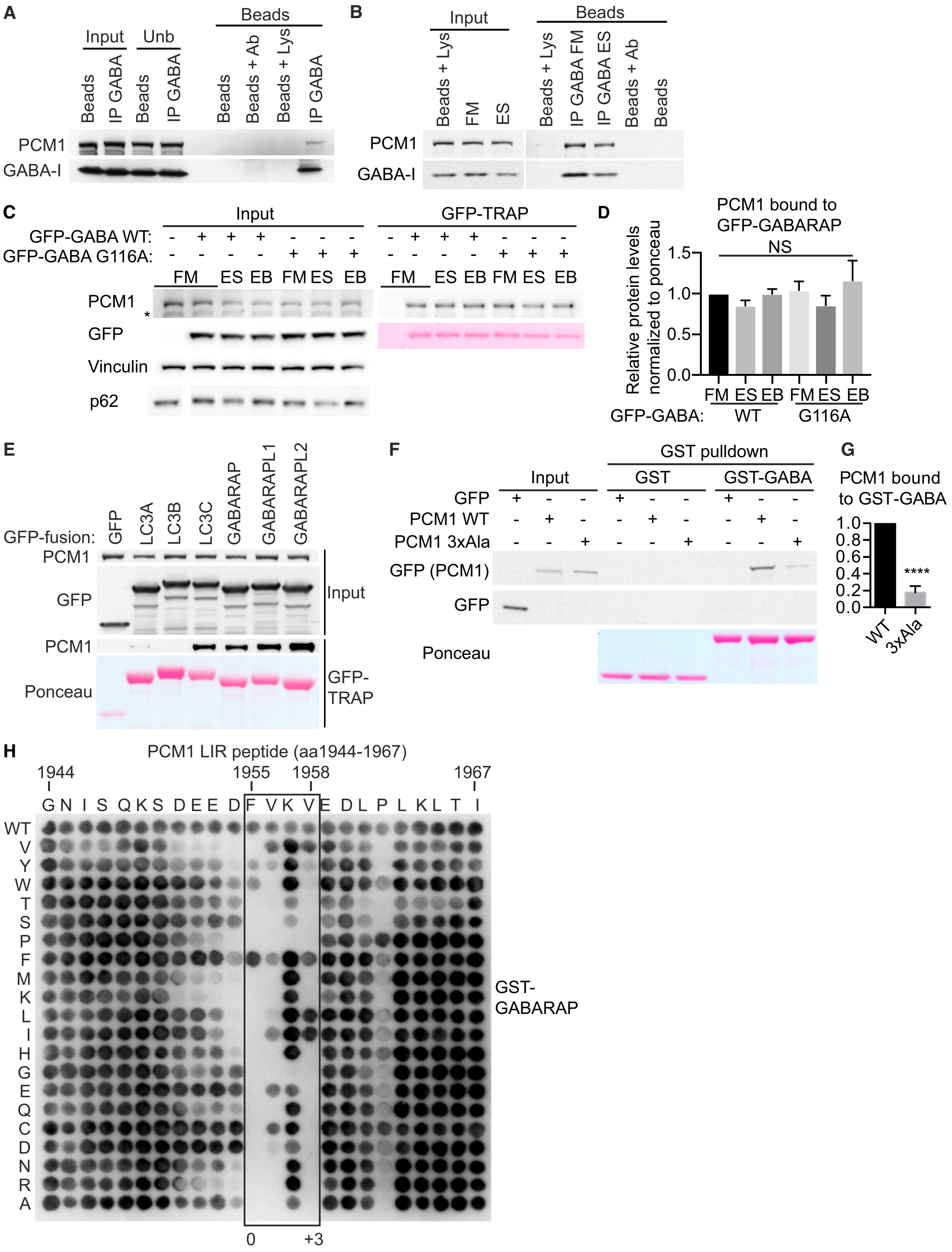

(legend on next page)

target cargoes into the autophagosome for degradation, and autophagy adaptors, which confer additional functionality but are not degraded by autophagy [10]. The functional differences between the LC3 and GABARAP subfamilies, and how these proteins are specifically regulated, are poorly understood. ATG8s have many interactors, often mediated through LC3-interacting region (LIR) motifs [11] present in cargo adaptors and receptors [10, 12]. How LIR-containing proteins specifically regulate ATG8s is a subject of intense investigation.

We have shown that GABARAP localizes at the pericentriolar material of the centrosome [13]. Centrosomes contain a pair of centrioles embedded in a matrix of coiled-coil proteins called the pericentriolar material (PCM) [14], which also contains microtubule-nucleating factors that enable the centrosome to function as a microtubule-organizing center. Electron-dense granules called centriolar satellites (CSs) surround the centrosome and are transported along microtubules [15]. PCM1 is the archetypal CS marker protein providing the structural scaffold for CSs [15, 16]. PCM1 is a large (~230 kDa) coiled-coil-containing protein that self-oligomerizes and binds other CS proteins, such as the E3 ligase Mib1 [17–19]. CS proteins colocalize with and bind PCM1 and require PCM1 for their pericentrosomal localization, and distinct populations of CS exist comprised of different proteins [15]. However, the functional significance of this is poorly understood.

Centrosomal GABARAP traffics to forming autophagosomes during starvation [13]. Importantly, this demonstrates a centrosome-autophagosome crosstalk. How centrosomal proteins regulate the transport of GABARAP from the centrosome to autophagosomes and GABARAP-mediated autophagy is unknown. Here, we show that GABARAP, but not LC3B, directly binds to the CS protein PCM1 through a LIR motif. GABARAP is found on a subset of peripheral CS, and its localization at the centrosome is controlled by PCM1. PCM1 promotes the formation of GABARAP-positive (but not LC3B-positive) autophagosomes and colocalizes with autophagy markers. PCM1 protects GABARAP from proteasomal degradation mediated by ubiquitination by Mib1. These data suggest that PCM1-containing CSs stabilize centrosomal GABARAP and control its delivery to autophagosomes.

RESULTS

PCM1 Binds GABARAP through a LIR Motif

The CS protein PCM1 interacts with overexpressed LC3B, GABARAP, and GABARAPL2 (GATE-16) [20, 21]. In HEK293A cells, PCM1 co-immunoprecipitated with endogenous GABARAP (Figure 1A), and this complex is not affected by amino acid starvation to induce autophagosome formation (Figure 1B). PCM1-GFP-GABARAP interaction was independent of GABARAP lipidation as PCM1 bound equally to GFP-GABARAP and GFP-GABARAP G116A, a mutant which cannot be lipidated [22], in fed, starved (ES), or starved cells treated with Bafilomycin A1 (BAFA1) to prevent lysosomal degradation of autophagosomes (Figures 1C and 1D). PCM1 bound ATG8 family members LC3C/GABARAP/GABARAPL1/GABARAPL2 but poorly to LC3A/LC3B (Figure 1E). We searched for LIR motifs in the 2,016aa human PCM1 protein (Refseq NP_001302436) using iLIR [23]. One LIR motif (aa 1,953-EDFVKV-aa 1,958) near the C terminus of PCM1, within a region required to retain PCM1 at the centrosome [24, 25], is similar to the human ULK LIR motif (DDFVM/LV) [26, 27]. Mutation of the PCM1 LIR EDFVKV to EAAVKA (3xAla) reduced binding to glutathione S-transferase (GST)-GABARAP by more than 80% (Figures 1F and 1G). A mutational peptide array of the PCM1 LIR motif showed PCM1 has a canonical LIR motif that directly binds GST-GABARAP (Figure 1H). D1954, F1955, V1956, V1958, and P1962 were essential for LIR binding. This is based on the LIR core of 0(W/F/Y)xx(L/I/V)+3 with surrounding acidic residues [28].

PCM1 Colocalizes with GABARAP at the Pericentriolar Material and CSs

We next tested whether PCM1-GABARAP colocalized in cells. In Figure 2A, we confirm GABARAP colocalizes with the centriole and pericentriolar material (PCM) marker γ-tubulin [13]. GABARAP was on the PCM and not centrioles, as shown by correlative light and electron microscopy (CLEM) (Figure S1). In cells where the PCM and centrioles were juxtaposed, GABARAP was on the PCM rather than centrioles (Figure S1). In the electron microscopy (EM), the PCM is a round, amorphous, dense structure near the centrioles [14]. We observed non-membrane-bound (Triton X-100 resistant) electron-dense granules (most likely CSs [29]), surrounding and embedded in the PCM (Figure S1). Concordantly, confocal microscopy revealed PCM1 partially overlapping with γ-tubulin, as expected (Figure 2A) [29].

PCM1 and GABARAP do not require γ-tubulin to colocalize. In mitotic cells, they colocalized to a γ-tubulin negative structure (Figure S2A) but much less at the spindle poles [13, 30]. In addition, GABARAP and PCM1 colocalized on a small number of peripherally distributed γ-tubulin-negative CSs (PCM1 puncta) (Figure 2A). Small interfering RNA (siRNA) depletion of PCM1 reduced the staining of peripheral PCM1 puncta and centrosomal clusters of PCM1, with two different PCM1 antibodies used here for immunofluorescence (Figure S2B). Colocalization

Figure 1. PCM1 Directly Binds GABARAP through a LIR Motif

(A) Anti-GABARAP immunoprecipitation from HEK293A cells and immunoblot. Beads + Ab, anti-GABARAP antibody with protein G beads; beads + Lys, HEK293A lysate with protein G beads.

(B) HEK293A cells in full medium (FM) or EBSS (ES) for 2 hr prior to lysis, followed by treatment as in (A).

(C) HEK293A cells expressing indicated constructs in FM, ES, or EBSS + BAFA (EB) for 2 hr prior to lysis and GFP-TRAP. GFP-GABA, GFP-GABARAP; WT, wild-type.

(D) Statistical analysis of (C); one-way ANOVA. NS, non-significant.

(E) GFP-TRAP of HEK293A cells expressing the indicated GFP-ATG8 constructs and immunoblot.

(F) HEK293A cells expressing the indicated GFP-tagged constructs incubated with GST or GST-GABARAP beads and immunoblotted. 3xAla, LIR mutant.

(G) Statistical analysis of (F); unpaired Student's t test; mean ± SEM; n = 3. ****$p \leq 0.0001$.

(H) 24-mer array of PCM1 peptides covering the LIR motif incubated with GST-GABARAP and immunoblot. Each amino acid position was substituted for every other amino acid.

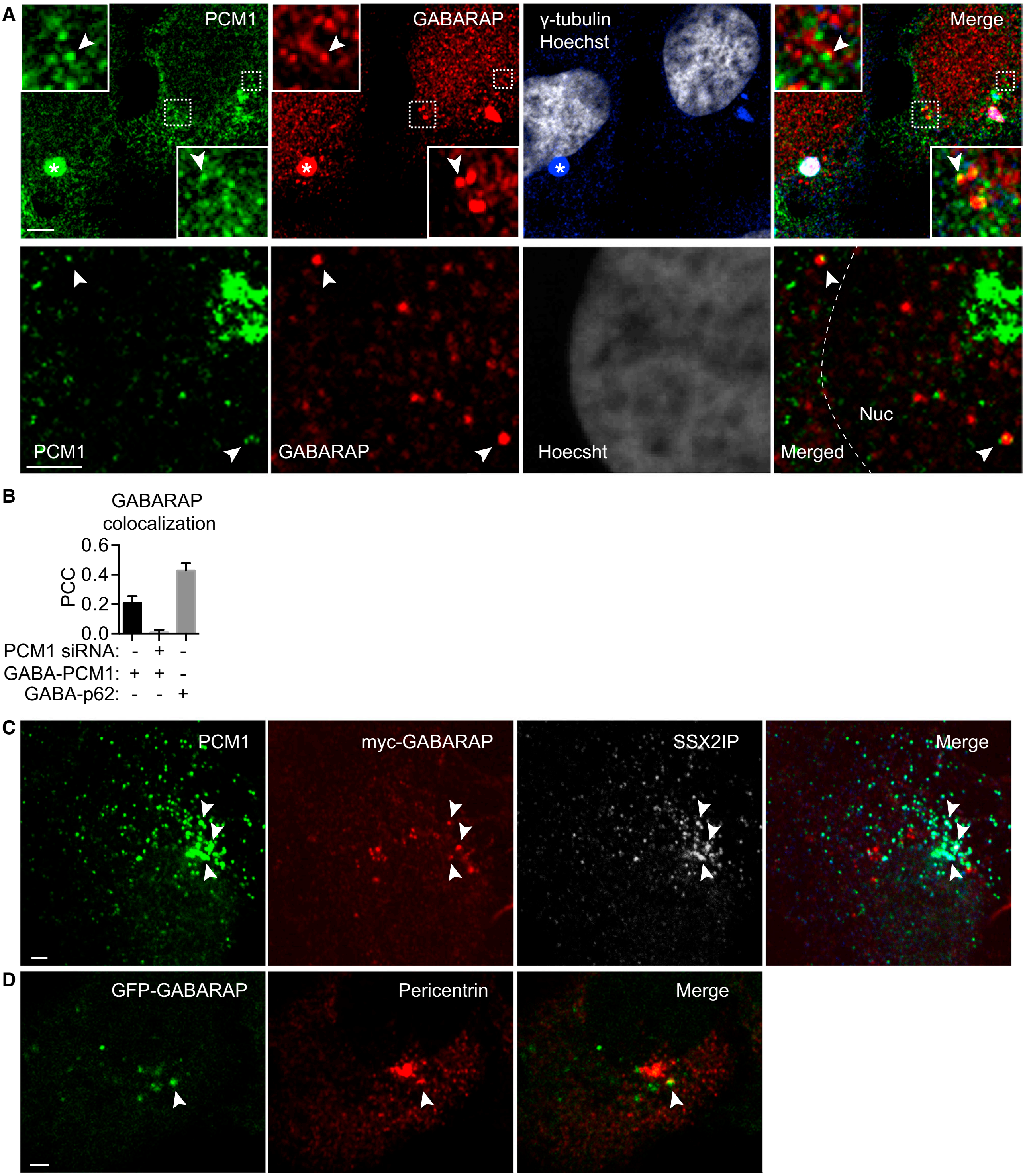

Figure 2. PCM1 and GABARAP Colocalize at the Centrosome and Peripheral CSs

(A) HEK293A cells starved for 2 hr in EBSS, fixed and labeled with the mouse anti-PCM1, rabbit anti-GABARAP, and goat anti-gamma tubulin antibodies. The scale bars represent (top) 5 μm and (bottom) 2 μm. Arrowheads and insets show PCM1-GABARAP colocalization. *GABARAP-PCM1 at the PCM. Nuc, nucleus.

(B) HEK293A cells starved for 2 hr in EBSS, fixed and labeled with the indicated antibodies. Pearson's correlation coefficient (PCC) was quantified between non-centrosomal GABARAP-PCM1 or GABARAP-p62 puncta, with and without PCM1 siRNA. Subcellular regions are quantified from two independent experiments.

(legend continued on next page)

analysis revealed a positive correlation between non-centrosomal PCM1-GABARAP puncta, which was lost after PCM1 knockdown (Figure 2B). Note, a stronger correlation between p62-GABARAP puncta was observed (Figure 2B), as expected because p62 is contained within autophagosomes.

We investigated whether GABARAP colocalized with other CS markers. myc-GABARAP partially colocalized with the CS marker and PCM1 interactor SSX2IP (Figure 2C) [31]. As expected, SSX2IP colocalized well with PCM1. GFP-GABARAP puncta partially colocalized with another PCM1 binding partner, pericentrin [32] (Figure 2D).

These data suggest that PCM1 and GABARAP interact in the PCM and that GABARAP is a novel component of a subset of CSs.

CSs Are Delivered to Forming Autophagosomes

Is PCM1, like GABARAP, recruited to autophagosomes? After 2 hr starvation to induce autophagy, a subset of PCM1-positive CSs colocalized with a range of autophagy markers: phagophore markers ATG9, ULK1 (Figure S2C), DFCP1, and WIPI2; autophagosome marker LC3B; and cargo protein p62 (Figures 3A and 3B). GFP-WIPI2b-positive phagophores contained PCM1 and GABARAP (Figure 3A). Interestingly, some PCM1 puncta were juxtaposed to ring-shaped GFP-DFCP1 omegasomes (Figure 3B). To confirm PCM1 is on early autophagosomes, we immunoisolated GFP-DFCP1-positive membranes. In addition to autophagy proteins ULK1 and GABARAP, CS proteins PCM1 and Mib1 [17, 18] associated with GFP-DFCP1 membranes (Figure 3C). Thus, PCM1-GABARAP-positive CSs are associated with autophagic structures and may be involved in autophagosome formation.

PCM1 Promotes GABARAP Centrosomal Localization

PCM1-positive CSs are involved in recruiting proteins to the centrosome [15]. PCM1 knockdown resulted in highly significant ($p \leq 0.0001$) reduction of GABARAP at the PCM (Figures 4A, 4B, and S3A) and a small increase in γ-tubulin. Centrosomal pericentrin was also reduced upon PCM1 depletion (Figure S3B), as reported [25]. Whereas no large frequency shifts in the brightness of γ-tubulin signals occurred upon PCM1 depletion, there was an increase in the number of centrosomes with weak GABARAP signal (Figure S3A).

Does PCM1-GABARAP colocalization require the LIR motif of PCM1 (Figures 1F–1H)? Overexpression of GFP-PCM1 wild-type (WT) revealed GFP-PCM1 at the GABARAP-positive PCM as expected (Figure 4C). Peripheral GFP-PCM1 structures were positive for GABARAP and WIPI2. GFP-PCM 3xAla also localized at the PCM with GABARAP; however, peripheral GABARAP-WIPI2 puncta were negative for GFP-PCM1 3xAla (Figure 4C). In contrast, peripheral non-centrosomal puncta of both GFP-PCM1 WT and 3xAla colocalized with the PCM1-interactor pericentrin (Figure S3C). Thus, the C-terminal LIR of PCM1 is required for GABARAP (but not pericentrin) recruitment to GFP-PCM1 puncta. These data suggest GABARAP is recruited to CS by PCM1 binding and PCM1 is involved in recruitment of GABARAP to the PCM.

CS GABARAP Is Not Golgi Associated

We showed that GABARAP binds the *cis*-Golgi protein GM130 [13]. Upon nocodazole treatment to depolymerize microtubules and disperse the Golgi away from the centrosome, GABARAP was on peripheral GM130 puncta [13]. We used nocodazole to disperse the Golgi and centrosomal CSs and found little colocalization between GM130 and GFP-PCM1 puncta (Figure S3D). We observed partial colocalization between GFP-PCM1-GABARAP and GABARAP-GM130 (Figure 4D), but very few structures were positive for all three proteins. These data suggest that Golgi-associated GABARAP is separate from CS-associated GABARAP.

PCM1 Regulates GABARAP Degradation and Autophagy

A subpopulation of PCM1-positive CSs are at autophagosome formation sites (Figures 3 and S2C). To determine whether PCM1 depletion affects autophagosome formation, starvation-induced WIPI2, GABARAP, LC3B, and p62 puncta were counted in HEK293A cells (Figures 4E and S4). Whereas WIPI2, GABARAP, and p62 puncta were increased after PCM1 knockdown, no significant effect on LC3B puncta was seen. Moreover, the number of GABARAP-p62 double-positive autophagosomes increased (Figure 4F). This suggests PCM1 regulates GABARAP-positive autophagosome formation.

Plk4 promotes phosphorylation of PCM1 at S372 [33]. S372 phosphorylation, which occurs during G1 of the cell cycle, promotes PCM1 dimerization and interaction with CS proteins. Compared to WT, PCM1 S372E phosphomimetic mutant exhibits reduced CS motility and increased clustering, whereas the S372A mutant has a more dispersed phenotype [33]. To determine whether this regulatory phosphorylation exerts an effect on GABARAP autophagosome formation, we expressed GFP-PCM1 WT, GFP-PCM1 S372A, or S372E and measured the formation of GABARAP-WIPI2 double-positive autophagosomes with BAFA1 present to prevent lysosomal degradation of autophagosomes. The number of GABARAP-WIPI2-positive autophagosomes was attenuated when PCM1 S372 was mutated to either E/A (Figure 4G). This suggests PCM1 S372 is important for GABARAP autophagosome formation and perhaps regulated by reversible phosphorylation events.

PCM1 is not required for rapamycin-induced autophagy [21]. Concordantly, PCM1 knockdown had no effect on LC3B lipidation or flux through the lysosome as measured by accumulation of lipidated LC3B (LC3-II) in the presence of BAFA1 (Figures 5A and 5B). PCM1 levels were not altered by autophagy, as there was no change in Earle's balanced salt solution (EBSS) compared to EBSS with BAFA1 (Figures 5A and 5B). However, we observed a decrease in both unlipidated and lipidated GABARAP (GABARAP-I and GABARAP-II, respectively),

(C) HEK293A cells expressing myc-GABARAP, starved for 2 hr in EBSS, and then fixed and labeled with rabbit anti-PCM1, mouse anti-myc, and goat anti-SSX2IP antibodies; scale bar, 2 μm.
(D) HEK293 Flp-In T-Rex cells stably expressing inducible GFP-GABARAP, starved for 2 hr in EBSS, were fixed and labeled with rabbit anti-pericentrin antibodies. The scale bar represents 2 μm. In (C) and (D), arrowheads show GABARAP colocalization with CS markers.
See also Figures S1 and S2.

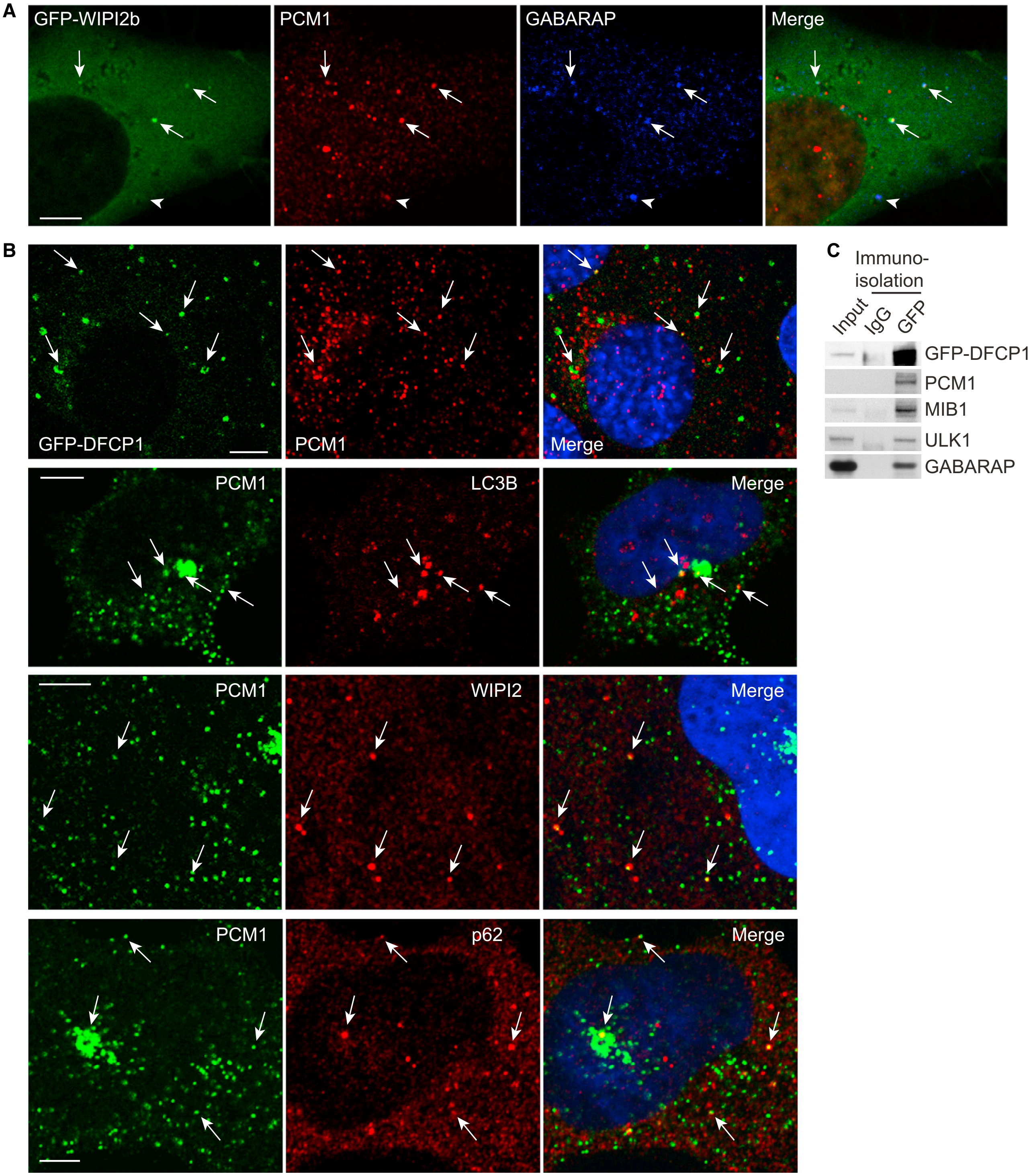

Figure 3. CSs Are Found at Sites of Autophagosome Formation

(A) HEK293A cells stably expressing GFP-WIPI2b starved for 2 hr in EBSS, fixed and labeled with mouse anti-PCM1 and rabbit anti-GABARAP antibodies. The scale bars represent 5 μm. Arrows indicate triple colocalization; arrowhead indicates GABARAP-PCM1 double colocalization.

(B) HEK293A cells, or MEF cells stably expressing GFP-DFCP1, starved for 2 hr in EBSS, fixed and labeled with the indicated antibodies. The scale bars represent 5 μm. Arrows indicate colocalization or points of contact (GFP-DFCP1) between PCM1 and autophagy markers. Hoechst DNA staining is shown in blue in the merge. In all panels, rabbit anti-PCM1 antibody was used; mouse anti-WIPI2 or LC3B and guinea pig anti-p62 antibodies were used in the remaining panels.

(C) HEK293 cells stably expressing GFP-DFCP1 starved for 2 hr in EBSS followed by immunoisolation with control immunoglobulin G (IgG) or anti-GFP and immunoblot analysis with the indicated antibodies.

See also Figure S2.

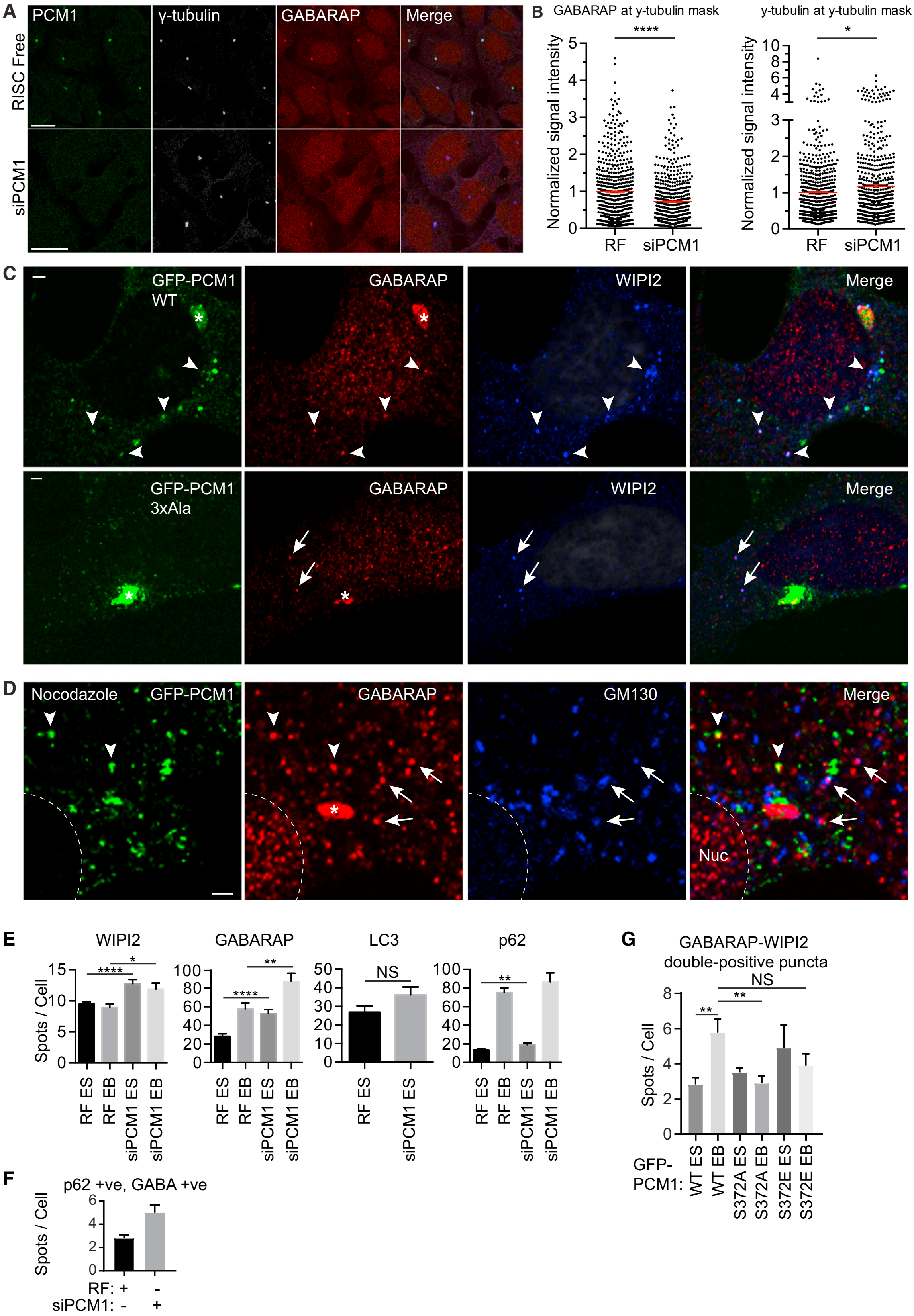

(legend on next page)

indicating a total decrease in GABARAP protein levels (Figures 5C–5E). Autophagy cargoes p62 and NBR1 were also decreased (Figures 5A and 5B). In addition, p62 and GABARAP protein levels increased upon GFP-PCM1 overexpression (Figures 5F and 5G). This suggests a specific effect of the PCM1 protein on GABARAP/p62 protein levels.

The LIR motif in PCM1 binds to (Figures 1F–1H) and localizes GABARAP (Figure 4C) to CS. We asked whether the LIR motif was also required for the stabilization of GABARAP seen after overexpression of GFP-PCM1. After siRNA depletion of PCM1, GABARAP levels were rescued by expression of GFP-PCM1 WT, but not by GFP-PCM1 3xAla (Figures 5H and 5I). These data suggest that direct binding of PCM1 to GABARAP stabilizes GABARAP protein levels.

We assessed the degradation rate of GABARAP in PCM1 knockout cells (Figures 6A and 6B). In single-guide (sgRNA) control RPE-1 cells, more than 50% of GABARAP remained during 8 hr cycloheximide treatment to inhibit translation. Without PCM1, the rate of GABARAP degradation was enhanced, suggesting that PCM1 stabilizes GABARAP. PCM1-controlled GABARAP degradation may occur through the proteasomal or lysosomal pathways. In HEK293A cells, we combined cycloheximide treatment with MG132, an inhibitor of the proteasome, or BAFA1 (Figures 6C and 6D). Proteasomal inhibition was confirmed by the accumulation of polyubiquitinated proteins after MG132 treatment (Figure 6C). Surprisingly, GABARAP turnover is inhibited by MG132 or BAFA1, suggesting that the proteasome degrades GABARAP to a similar extent as autophagy during basal conditions. However, after PCM1 depletion, significantly more GABARAP was degraded through the proteasome than the lysosome (Figure 6D). Altogether, these data suggest PCM1 specifically regulates GABARAP autophagosome formation and autophagic flux of cargoes and stabilizes GABARAP from proteasomal degradation.

GABARAP Instability and Ubiquitination Is Driven by Mib1

PCM1 binds and stabilizes GABARAP from proteasomal degradation, and this may occur at the PCM, CS, and sites of autophagosome formation (Figures 1, 2, 3, 4, and 5). PCM1 sequesters the CS E3 ligase Mib1 [17, 18]. PCM1 depletion results in increased Mib1 levels and relocalization of Mib1 from CS to centrioles, where it ubiquitinates and destabilizes centrosomal proteins [17]. In HEK293A cells, Mib1 protein levels increased after PCM1 depletion, as expected, concomitant with a decrease in GABARAP and p62 levels (Figure S5A). FLAG-Mib1 expression decreased PCM1 levels as expected [17] but also GABARAP levels, suggesting that Mib1 interacts with GABARAP (Figures 7A and 7B). Mib1 bound to GST-GABARAP, and FLAG-Mib1 co-immunoprecipitated with GABARAP (Figures S5B and S5C). Additionally, endogenous Mib1 and GABARAP co-immunoprecipitated (Figure 7C). GST-GABARAP bound both WT FLAG-Mib1 and catalytically inactive Mib1 (Figure S5D). By mapping experiments, GST-GABARAP interacted with aa 1–429 of Mib1 (Figure 7D), which contains the N-terminal zinc finger (Figure S5E). Substrates of Mib1-mediated ubiquitination are known to interact with this region [34, 35].

We next investigated whether Mib1 promotes GABARAP ubiquitination. Mib1 promoted the mono-, di-, tri-, and poly-ubiquitination of GFP-GABARAP (Figures 7E, S5F, and S5G). GFP-GABARAP was more readily ubiquitinated than GFP-LC3B (Figure 7F). We confirmed GFP-GABARAP mono-, di-, tri-, and poly-ubiquitination with an anti-ubiquitin antibody after washing immunoprecipitated GFP-GABARAP with denaturing 8 M urea and 1% SDS (Figure 7G). Mib1 promotes both K48- and K63-linked ubiquitination [36, 37]. Di-, tri-, and poly-ubiquitination of GFP-GABARAP occurred through K48-linked (Figure 7H), but not K63-linked, ubiquitination (Figure S5H). K48 ubiquitin linkages are often associated with proteasomal degradation [38]. Large polyubiquitinated GFP-GABARAP conjugates (>300 kDa) were detected as K63 linked (Figure S5H). We detected a small population of di- and tri-ubiquitinated endogenous GABARAP (Figures 7I and 7J). Moreover, di- and tri-ubiquitinated GABARAP was depleted by GABARAP siRNA, suggesting that these were conjugates of GABARAP (Figure 7J). Finally, mass spectrometry revealed FLAG-Mib1-driven ubiquitination of GFP-GABARAP occurs at lysine 13 and 23 (Figures 7K, 7L and S5I–S5K).

DISCUSSION

Many organelles/structures regulate the formation of the autophagosome [21, 39–42]. The (non-ciliated) centrosome also

Figure 4. PCM1 Controls GABARAP Localization at the PCM, CS, and GABARAP Autophagosome Formation

(A) HEK293A cells were treated with RISC free (RF) or PCM1 siRNA for 72 hr, fixed, and labeled with the mouse anti-PCM1, rabbit anti-GABARAP, and goat anti-gamma tubulin antibodies. The scale bar represents 20 μm.

(B) Quantification of (A). Signal intensities at the pericentriolar material (γ-tubulin positive structures) were quantified and normalized to RF. Each measurement represents one centrosome. Statistical analysis using Mann-Whitney test; mean ± SEM; data from three independent experiments. ****$p \leq 0.0001$.

(C) HEK293A cells expressing GFP-PCM1 WT or 3xAla LIR mutant starved for 2 hr in EBSS, fixed, and labeled with rabbit anti-GABARAP and mouse anti-WIPI2 antibodies. The scale bars represent 2 μm. Arrowheads, triple colocalization; arrows, GABARAP-WIPI2 colocalization. *PCM1 and GABARAP at the PCM.

(D) HEK293A cells expressing GFP-PCM1 were treated with 50 μM nocodazole for 5 hr in total and starved for 2 hr in EBSS prior to fixation and labeling with rabbit anti-GABARAP and mouse anti-GM130 antibodies. Arrowheads, GFP-PCM1-GABARAP colocalization; arrows, GM130-GABARAP colocalization. *GABARAP at PCM. The scale bar represents 2 μm.

(E) HEK293A cells were treated with RF or PCM1 siRNA for 72 hr and then incubated in ES or in ES with bafilomycin A1 (EB) for 2 hr, fixed, and labeled with the indicated antibodies before confocal microscopy and quantification of intracellular puncta. Statistical analysis using unpaired Student's t test; mean ± SEM; *$p \leq 0.05$. Number of independent experiments: WIPI2, three; GABARAP, five; p62, three; and LC3, two.

(F) HEK293A cells were treated with RF or PCM1 siRNA for 72 hr and then incubated in EBSS for 2 hr and fixed before confocal microscopy and quantification of intracellular GABARAP-p62 double-positive puncta. Mean ± SEM two independent experiments.

(G) HEK293A cells expressing the indicated constructs were incubated in ES or in EB for 2 hr, fixed, and labeled with the indicated antibodies before confocal microscopy and quantification of intracellular puncta. Statistical analysis using unpaired Student's t test; mean ± SEM; **$p \leq 0.01$. Three independent experiments.

See also Figures S3 and S4.

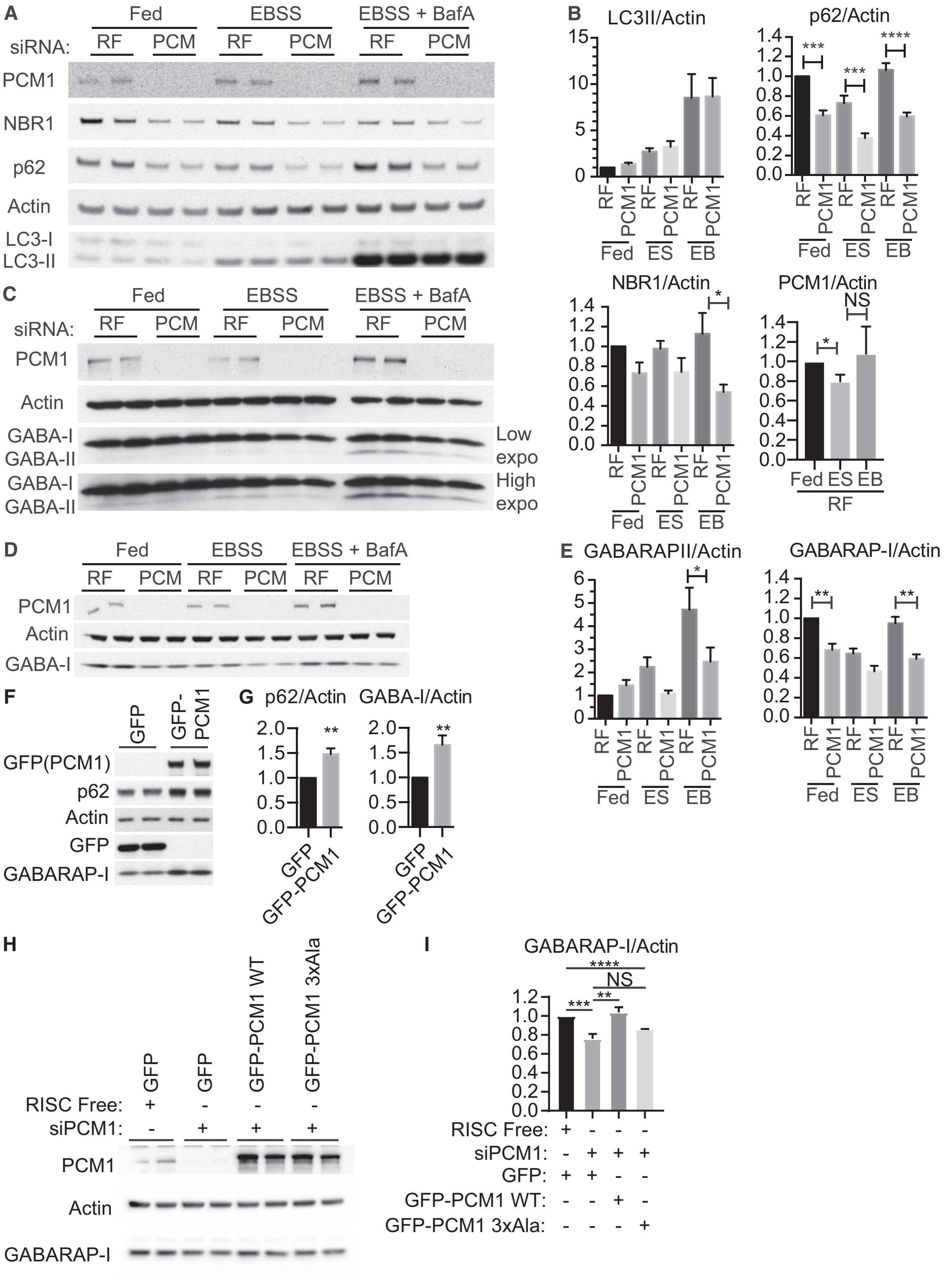

Figure 5. PCM1 Specifically Regulates GABARAP Protein Levels through Its LIR Motif

(A) HEK293A cells treated with RF or PCM1 siRNA incubated in full medium (FM) or EBSS with or without BAFA for 2 hr.

(B) Quantifications from (A). For LC3-II/actin, n = 3. For p62/actin, n = 5. For NBR1/actin, n = 5. For PCM1/actin, n = 5. Mean ± SEM. One-way ANOVA; *p $\leq$ 0.05.

(C and D) HEK293A cells treated with RF or PCM1 siRNA incubated in FM or EBSS with or without BAFA1 for 2 hr. In (C), GABARAP-I and GABARAP-II are resolved, whereas in (D) only GABARAP-I is resolved in the western blot.

(legend continued on next page)

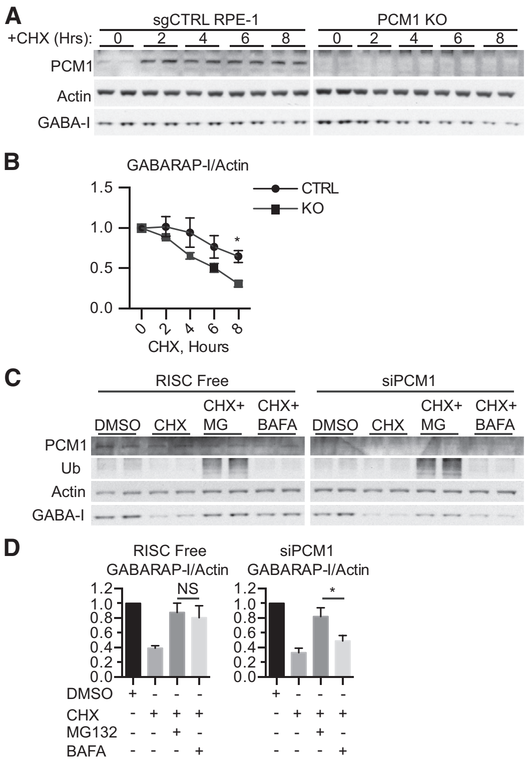

Figure 6. PCM1 Regulates GABARAP Proteasomal Degradation

(A) Control or PCM1 knockout RPE-1 cells were subjected to cycloheximide (CHX) treatment for the indicated number of hours prior to immunoblotting.

(B) Quantification of (A). Mean ± SEM; n = 3; unpaired Student's t test; *p $\leq$ 0.05.

(C) HEK293A cells treated with RF or PCM1 siRNA were incubated in DMSO, cycloheximide (CHX), MG132 (MG), and/or bafilomycin A1 (BAFA1) for 8 hr.

(D) Quantification of (C). Mean ± SEM; n = 3; one-way ANOVA; *p $\leq$ 0.05.

appears to regulate autophagy [13]. Here, we describe a role for CS in autophagy regulation, giving insight into the function and regulation of the enigmatic centrosomal pool of GABARAP and centrosome-autophagosome communication.

Our data suggest GABARAP is a novel component of a subset of CSs. CSs are not a homogeneous population, and whereas they have established functions, for example in ciliogenesis, the role of different types of CSs is poorly understood [15]. This study implicates PCM1-GABARAP-positive CSs in delivery to autophagosomes and autophagy regulation. We favor a model whereby GABARAP bound to CS is delivered to forming autophagosomes to perform its autophagic function. This is supported by our work showing delivery of centrosomal GABARAP to pre-existing autophagic structures upon starvation [13].

The role of basal autophagy in ciliogenesis in dividing cells is thought to be to degrade ciliogenesis activators (IFT20 and IFT88) [41, 43]. However, in non-dividing cells serum starved for 24 hr, the inhibitor of ciliogenesis, OFD1, a CS protein, is degraded by autophagy, but PCM1 is not [21]. In addition, we found that the slight decrease in PCM1 protein levels seen after 2 hr of amino acid starvation is not increased by bafilomycin A1 treatment (Figure 5B). Furthermore, the PCM1-GABARAP interaction is independent of amino acid levels (Figures 1B–1D). It is likely that GABARAP-PCM1 CS localization at autophagosomes is separate from ciliogenesis, as PCM1 colocalization with autophagosome markers was seen during 2 hr starvation, a timescale too short for significant primary cilia formation [44]. In addition, we found no evidence for a requirement of GABARAP for cilia formation (data not shown). However, we do not rule out the possibility that multiple ATG8 proteins act redundantly in ciliogenesis.

CSs are transported along microtubules [19, 25, 29], which could explain how GABARAP at the pericentriolar material could be transferred to distal forming autophagosomes. By immunofluorescence microscopy, GABARAP is enriched within the PCM compared to the adjacent CS. This is disrupted upon PCM1 knockdown (Figures 4A, 4B, and S3A). We also saw structures reminiscent of CSs embedded in the PCM by CLEM (Figure S1). Combined with the role of PCM1 in enhancing GABARAP stability (Figures 5 and 6), we propose that CSs regulate the recruitment and stabilization of GABARAP at the PCM.

PCM1 depletion has no effect on autophagy, as measured by LC3 lipidation alone (Figures 5A and 5B) [21]. However, PCM1 binds very weakly to LC3B in comparison to GABARAP (Figure 1E). PCM1-GABARAP binding is mediated through a canonical ULK-type LIR motif (Figures 1F–1H). PCM1 depletion results in destabilization of GABARAP, but not LC3B, and a reduction of proteins degraded by autophagy (p62 and NBR1) (Figures 5A–5E, 6A, and 6B). In addition, more GABARAP-positive autophagosomes are formed after PCM1 depletion, but LC3B-positive autophagosomes are unaffected (Figures 4E and S4). Based on our data, we suggest that GABARAP is held in an inactive (non-autophagic) state through direct binding to the PCM1 LIR motif on CSs. This would prevent GABARAP from recruiting LIR-containing proteins [11] or LIR-containing ATG proteins, such as ULK1 [13]. However, these CSs can be sent to forming autophagosomes to function in autophagy when required. In addition, stabilization and recruitment of GABARAP by CSs to the PCM maintains a reservoir of unlipidated non-autophagic GABARAP at the centrosome [13]. This pool is poised to contribute to autophagosome formation upon starvation. How this is fully regulated and the importance or function of centrosomal GABARAP remains to be demonstrated. In the absence of PCM1, GABARAP is destabilized through Mib1 activity (Figures 7 and S5) and GABARAP is released from PCM1, freeing up its LIR-binding pocket. This dysregulated GABARAP could then readily form autophagosomes, resulting in enhanced p62 and NBR1 degradation. Interestingly, Mib1 relocalizes from CSs to

(E) Quantifications from (C) and (D). For GABARAP-II/actin, n = 5. For GABARAP-I/actin, n = 3. Mean ± SEM. One-way ANOVA; *p $\leq$ 0.05.

(F) HEK293A cells expressing the indicated constructs were subjected to immunoblot.

(G) Quantification of (F). For p62/actin, n = 4. For GABARAP-I/actin, n = 5. Mean ± SEM; unpaired Student's t test; **p $\leq$ 0.01.

(H) HEK293A cells treated with RF or PCM1 siRNA (72 hr total) and transfected with the indicated siPCM1-resistant constructs (last 24 hr) and immunoblotted.

(I) Quantifications from (H). For RF+GFP, siPCM1+GFP, and siPCM1+GFP-PCM1 WT, six experiments are shown. For siPCM1+GFP-PCM1 3xAla, three experiments are shown. Mean ± SEM; unpaired Student's t test; **p $\leq$ 0.01.

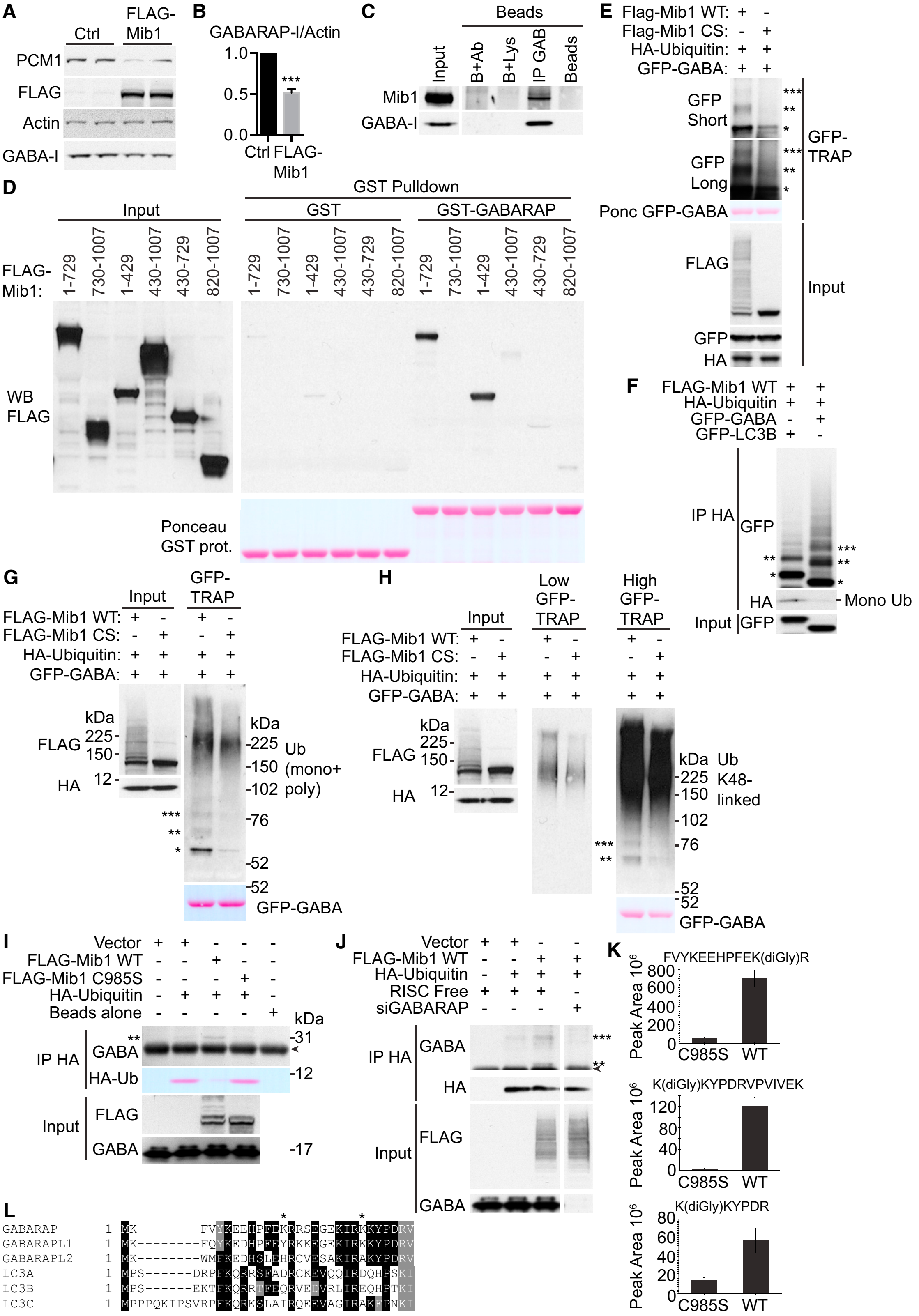

Figure 7. Mib1 E3 Ligase Interacts with and Destabilizes GABARAP and Promotes GABARAP Ubiquitination at Lys13 and Lys23

(A) HEK293A cells expressing FLAG-Mib1 or control vector for 48 hr were analyzed by immunoblot.

(B) Quantification of (A). Statistical analysis using unpaired Student's t test; mean ± SEM; n = 3; *p ≤ 0.001.

(legend continued on next page)

centrioles after PCM1 depletion [17], which places Mib1 in proximity for GABARAP ubiquitination.

This study contributes to efforts in the autophagy field to understand why ATG8 has undergone divergent evolution from yeast to human and the functional/regulatory differences between the six orthologs. Recently, GABARAPs, but not LC3s, were shown to be required for starvation-induced autophagy and also mitophagy [7, 45]. The full role of GABARAP-enriched autophagosomes is not known. Perhaps PCM1 has a role in GABARAP-specific autophagy, such as in damaged mitochondrial clearance.

Surprisingly, we found that GABARAP is efficiently degraded through both the proteasome and lysosome (Figures 6A–6D). The rate of GABARAP turnover in RPE-1 cells was enhanced in the absence of PCM1 (Figures 6A and 6B). In HEK293A cells, GABARAP is constitutively turned over, and proportionately more GABARAP is degraded through the proteasome than the lysosome upon PCM1 depletion (Figures 6C and 6D). Nonetheless, we saw an increase of GABARAP-positive autophagosomes by immunofluorescence microscopy (Figures 4E and S4), suggesting increased autophagosome formation.

GABARAP was thought to not be degraded by the proteasome [46]; however, recently, ATG4B binding GABARAP through a LIR motif was shown to stabilize GABARAP from proteasomal degradation [47]. As PCM1 also regulates GABARAP protein levels through a LIR motif (Figures 5H and 5I), perhaps LIR binding to GABARAP is a general mechanism of stabilization. The PCM1-binding CS E3 ligase Mib1 [17, 18], which is no longer sequestered by PCM1 after PCM1 depletion, destabilizes GABARAP, most likely through K48-linked ubiquitination of GABARAP, which probably occurs at K13 and K23. In support, ubiquitination of endogenous GABARAP at K13 and K23 has been reported using mass spectrometry [48]. Our data suggest this destabilization occurs through proteasomal degradation of GABARAP. Interestingly, LC3B is a much poorer target for ubiquitination promoted by Mib1 than GABARAP (Figure 7F). This could be related to preferential binding of GABARAP to PCM1 and GABARAP localization to the pericentriolar material, both of which would bring GABARAP in proximity to Mib1. The ubiquitination of GABARAP within an N-terminal helix is also interesting, as this region is less well conserved between ATG8 orthologs (see Figure 7L, and [6]), and so ubiquitination at these sites may provide a method of specific regulation of ATG8s.

In conclusion, our results shed light on the role of CSs in centrosomal regulation of starvation-induced autophagy. We hypothesize that the centrosomal pool of GABARAP may be a storage pool. At resting state, autophagy proteins are already translated and poised to act during acute autophagy stimulation. Whereas several core autophagy proteins exert non-autophagic functions [49], it may be that their preservation at a resting state location facilitates rapid autophagosome formation induced by an acute signal, such as amino acid starvation.

STAR★METHODS

Detailed methods are provided in the online version of this paper and include the following:

- KEY RESOURCES TABLE
- CONTACT FOR REAGENT AND RESOURCE SHARING
- EXPERIMENTAL MODEL AND SUBJECT DETAILS
  - Cell lines and culture
  - Organisms for recombinant protein expression
- METHOD DETAILS
  - siRNA/DNA transfection and antibodies
  - Western Blotting
  - Immunoprecipitation
  - Immunoisolation of GFP-DFCP1 membranes
  - GST pulldowns
  - Peptide Arrays and GST Overlay Assay
  - Ubiquitination assays
  - Ubiquitination mass spectrometry
  - Confocal microscopy
  - Confocal data quantification
  - Correlative Light and Electron Microscopy
  - Primers used in this study
- QUANTIFICATION AND STATISTICAL ANALYSIS
- DATA AND SOFTWARE AVAILABILITY

(C) Anti-GABARAP immunoprecipitate from HEK293A cells analyzed by immunoblotting. Ab, anti-GABARAP antibody. Lys, HEK293A lysate.
(D) HEK293A cells expressing FLAG-tagged constructs were incubated with recombinant GST or GST-GABARAP beads and immunoblotted.
(E) GFP-TRAP of HEK293A cells expressing the indicated constructs and immunoblot. Immunoprecipitates were stringently washed in denaturing buffer. CS, C985S; GAB, GABARAP; Ponc, Ponceau S. Short and long exposures are shown. *, **, ***, mono-, di-, and tri-ubiquitinated GFP-GABARAP, respectively.
(F) Immunoprecipitation of U2OS cells expressing the indicated constructs lysed in boiling SDS buffer and immunoblot. Free ubiquitin and *, **, ***, mono-, di-, and tri-ubiquitinated GFP-LC3B/GABARAP are indicated, respectively.
(G) GFP-TRAP of HEK293A cells expressing the indicated constructs and immunoblot. Immunoprecipitates were washed as in (E).
(H) See (G). Low and high exposures are shown.
(I) Immunoprecipitation of HEK293A cells expressing the indicated constructs and immunoblot. Cells were treated with MG132 for 5 hr prior to lysis in TNTE buffer (20 mM Tris, pH 7.4, 150 mM NaCl, 0.5% w/v Triton X-100, 5 mM EDTA) + N-ethylmaleimide. Diubiquitinated GABARAP is indicated with **. Immunoglobulin light chain is indicated with an arrow.
(J) Immunoprecipitation of HEK293A cells treated with RF or GABARAP siRNA for 72 hr and expressing the indicated constructs and immunoblot. Cells were treated with MG132 for 5 hr prior to lysis in TNTE buffer + N-ethylmaleimide. Di- and tri-ubiquitinated GABARAP is indicated with ** and ***, respectively. Immunoglobulin light chain is indicated with an arrow.
(K) Two GABARAP ubiquitination sites, lysine 13 (K13) and lysine 23 (K23), were identified by mass spectrometry on three different peptides. (Top) Comparison of peak areas for the FVYKEEHPFEK(diGly)R peptide containing K13 ubiquitination site (n = 3 measurements) is shown. (Middle and bottom) The K23 ubiquitination site was detected as two different peptides as a result of missed cleavage. Quantification of peptides K(diGly)KYPDRVPVIVEK (middle) and K(diGly)KYPDR (bottom) showed significantly lower abundance in C985S mutant compared to the WT.
(L) Conservation of GABARAP K13 and K23 (*) between ATG8 orthologs.
See also Figure S5.

SUPPLEMENTAL INFORMATION

Supplemental Information includes five figures and can be found with this article online at http://dx.doi.org/10.1016/j.cub.2017.06.021.

AUTHOR CONTRIBUTIONS

Conceptualization, J.J., M.R., and S.A.T.; Methodology, J.J., M.R., D.J., M.W., N.O., V.E., A.P.S., and H.B.J.J.; Investigation, J.J., M.R., D.J., M.W., E.C., V.E., and H.B.J.J.; Writing – Original Draft, J.J.; Writing – Reviewing and Editing, J.J., B.D.D., M.R., and S.A.T.; Funding Acquisition, S.A.T.; Resources, A.P.S., N.O., and B.D.D.; Supervision, J.J. and S.A.T.

ACKNOWLEDGMENTS

We thank Sylvie Urbé (University of Liverpool) for advice and suggestions regarding the ubiquitination experiment, Jason Berndt (Howard Hughes Medical Institute) for the Mib1 constructs, Takashi Toda (University of Hiroshima) for EGFP-PCM1, Noboru Mizushima (University of Tokyo) for GFP-DFCP1 mouse embryonic fibroblasts (MEFs), and Matthew Renshaw (The Francis Crick Institute) for help with microscopy data analysis. This work was supported by the Francis Crick Institute, which receives its core funding from Cancer Research UK (FC001187 and FC001999); the UK Medical Research Council (FC001187 and FC001999); and the Wellcome Trust (FC001187 and FC001999). The work in B.D.D.'s laboratory was supported by funds from the NIH (HD069647 and 9R01 GM 120776-05A1).

Received: December 2, 2016
Revised: May 5, 2017
Accepted: June 8, 2017
Published: July 13, 2017

REFERENCES

1. Carlsson, S.R., and Simonsen, A. (2015). Membrane dynamics in autophagosome biogenesis. J. Cell Sci. *128*, 193–205.
2. Karanasios, E., Walker, S.A., Okkenhaug, H., Manifava, M., Hummel, E., Zimmermann, H., Ahmed, Q., Domart, M.-C., Collinson, L., and Ktistakis, N.T. (2016). Autophagy initiation by ULK complex assembly on ER tubulovesicular regions marked by ATG9 vesicles. Nat. Commun. *7*, 12420.
3. Dooley, H.C., Razi, M., Polson, H.E., Girardin, S.E., Wilson, M.I., and Tooze, S.A. (2014). WIPI2 links LC3 conjugation with PI3P, autophagosome formation, and pathogen clearance by recruiting Atg12-5-16L1. Mol. Cell *55*, 238–252.
4. Orsi, A., Razi, M., Dooley, H.C., Robinson, D., Weston, A.E., Collinson, L.M., and Tooze, S.A. (2012). Dynamic and transient interactions of Atg9 with autophagosomes, but not membrane integration, are required for autophagy. Mol. Biol. Cell *23*, 1860–1873.
5. Lamb, C.A., Nühlen, S., Judith, D., Frith, D., Snijders, A.P., Behrends, C., and Tooze, S.A. (2016). TBC1D14 regulates autophagy via the TRAPP complex and ATG9 traffic. EMBO J. *35*, 281–301.
6. Slobodkin, M.R., and Elazar, Z. (2013). The Atg8 family: multifunctional ubiquitin-like key regulators of autophagy. Essays Biochem. *55*, 51–64.
7. Nguyen, T.N., Padman, B.S., Usher, J., Oorschot, V., Ramm, G., and Lazarou, M. (2016). Atg8 family LC3/GABARAP proteins are crucial for autophagosome-lysosome fusion but not autophagosome formation during PINK1/Parkin mitophagy and starvation. J. Cell Biol. *215*, 857–874.
8. Weidberg, H., Shpilka, T., Shvets, E., Abada, A., Shimron, F., and Elazar, Z. (2011). LC3 and GATE-16 N termini mediate membrane fusion processes required for autophagosome biogenesis. Dev. Cell *20*, 444–454.
9. Weidberg, H., Shvets, E., Shpilka, T., Shimron, F., Shinder, V., and Elazar, Z. (2010). LC3 and GATE-16/GABARAP subfamilies are both essential yet act differently in autophagosome biogenesis. EMBO J. *29*, 1792–1802.
10. Stolz, A., Ernst, A., and Dikic, I. (2014). Cargo recognition and trafficking in selective autophagy. Nat. Cell Biol. *16*, 495–501.
11. Pankiv, S., Clausen, T.H., Lamark, T., Brech, A., Bruun, J.-A., Outzen, H., Øvervatn, A., Bjørkøy, G., and Johansen, T. (2007). p62/SQSTM1 binds directly to Atg8/LC3 to facilitate degradation of ubiquitinated protein aggregates by autophagy. J. Biol. Chem. *282*, 24131–24145.
12. Zaffagnini, G., and Martens, S. (2016). Mechanisms of selective autophagy. J. Mol. Biol. *428* (9 Pt A), 1714–1724.
13. Joachim, J., Jefferies, H.B.J., Razi, M., Frith, D., Snijders, A.P., Chakravarty, P., Judith, D., and Tooze, S.A. (2015). Activation of ULK kinase and autophagy by GABARAP trafficking from the centrosome is regulated by WAC and GM130. Mol. Cell *60*, 899–913.
14. Woodruff, J.B., Wueseke, O., and Hyman, A.A. (2014). Pericentriolar material structure and dynamics. Philos. Trans. R. Soc. Lond. B Biol. Sci. *369*, 20130459.
15. Hori, A., and Toda, T. (2017). Regulation of centriolar satellite integrity and its physiology. Cell. Mol. Life Sci. *74*, 213–229.
16. Tollenaere, M.A.X., Mailand, N., and Bekker-Jensen, S. (2015). Centriolar satellites: key mediators of centrosome functions. Cell. Mol. Life Sci. *72*, 11–23.
17. Wang, L., Lee, K., Malonis, R., Sanchez, I., and Dynlacht, B.D. (2016). Tethering of an E3 ligase by PCM1 regulates the abundance of centrosomal KIAA0586/Talpid3 and promotes ciliogenesis. eLife *5*, 12950.
18. Villumsen, B.H., Danielsen, J.R., Povlsen, L., Sylvestersen, K.B., Merdes, A., Beli, P., Yang, Y.G., Choudhary, C., Nielsen, M.L., Mailand, N., and Bekker-Jensen, S. (2013). A new cellular stress response that triggers centriolar satellite reorganization and ciliogenesis. EMBO J. *32*, 3029–3040.
19. Kubo, A., and Tsukita, S. (2003). Non-membranous granular organelle consisting of PCM-1: subcellular distribution and cell-cycle-dependent assembly/disassembly. J. Cell Sci. *116*, 919–928.
20. Behrends, C., Sowa, M.E., Gygi, S.P., and Harper, J.W. (2010). Network organization of the human autophagy system. Nature *466*, 68–76.
21. Tang, Z., Lin, M.G., Stowe, T.R., Chen, S., Zhu, M., Stearns, T., Franco, B., and Zhong, Q. (2013). Autophagy promotes primary ciliogenesis by removing OFD1 from centriolar satellites. Nature *502*, 254–257.
22. Kabeya, Y., Mizushima, N., Yamamoto, A., Oshitani-Okamoto, S., Ohsumi, Y., and Yoshimori, T. (2004). LC3, GABARAP and GATE16 localize to autophagosomal membrane depending on form-II formation. J. Cell Sci. *117*, 2805–2812.
23. Kalvari, I., Tsompanis, S., Mulakkal, N.C., Osgood, R., Johansen, T., Nezis, I.P., and Promponas, V.J. (2014). iLIR: A web resource for prediction of Atg8-family interacting proteins. Autophagy *10*, 913–925.
24. Insolera, R., Shao, W., Airik, R., Hildebrandt, F., and Shi, S.H. (2014). SDCCAG8 regulates pericentriolar material recruitment and neuronal migration in the developing cortex. Neuron *83*, 805–822.
25. Dammermann, A., and Merdes, A. (2002). Assembly of centrosomal proteins and microtubule organization depends on PCM-1. J. Cell Biol. *159*, 255–266.
26. Kraft, C., Kijanska, M., Kalie, E., Siergiejuk, E., Lee, S.S., Semplicio, G., Stoffel, I., Brezovich, A., Verma, M., Hansmann, I., et al. (2012). Binding of the Atg1/ULK1 kinase to the ubiquitin-like protein Atg8 regulates autophagy. EMBO J. *31*, 3691–3703.
27. Alemu, E.A., Lamark, T., Torgersen, K.M., Birgisdottir, A.B., Larsen, K.B., Jain, A., Olsvik, H., Øvervatn, A., Kirkin, V., and Johansen, T. (2012). ATG8 family proteins act as scaffolds for assembly of the ULK complex: sequence requirements for LC3-interacting region (LIR) motifs. J. Biol. Chem. *287*, 39275–39290.
28. Johansen, T., and Lamark, T. (2011). Selective autophagy mediated by autophagic adapter proteins. Autophagy *7*, 279–296.
29. Kubo, A., Sasaki, H., Yuba-Kubo, A., Tsukita, S., and Shiina, N. (1999). Centriolar satellites: molecular characterization, ATP-dependent movement toward centrioles and possible involvement in ciliogenesis. J. Cell Biol. *147*, 969–980.
30. Balczon, R., Bao, L., and Zimmer, W.E. (1994). PCM-1, a 228-kD centrosome autoantigen with a distinct cell cycle distribution. J. Cell Biol. *124*, 783–793.

CellPress

31. Klinger, M., Wang, W., Kuhns, S., Bärenz, F., Dräger-Meurer, S., Pereira, G., and Gruss, O.J. (2014). The novel centriolar satellite protein SSX2IP targets Cep290 to the ciliary transition zone. Mol. Biol. Cell *25*, 495–507.

32. Li, Q., Hansen, D., Killilea, A., Joshi, H.C., Palazzo, R.E., and Balczon, R. (2001). Kendrin/pericentrin-B, a centrosome protein with homology to pericentrin that complexes with PCM-1. J. Cell Sci. *114*, 797–809.

33. Hori, A., Barnouin, K., Snijders, A.P., and Toda, T. (2016). A non-canonical function of Plk4 in centriolar satellite integrity and ciliogenesis through PCM1 phosphorylation. EMBO Rep. *17*, 326–337.

34. Berndt, J.D., Aoyagi, A., Yang, P., Anastas, J.N., Tang, L., and Moon, R.T. (2011). Mindbomb 1, an E3 ubiquitin ligase, forms a complex with RYK to activate Wnt/β-catenin signaling. J. Cell Biol. *194*, 737–750.

35. Itoh, M., Kim, C.H., Palardy, G., Oda, T., Jiang, Y.J., Maust, D., Yeo, S.Y., Lorick, K., Wright, G.J., Ariza-McNaughton, L., et al. (2003). Mind bomb is a ubiquitin ligase that is essential for efficient activation of Notch signaling by Delta. Dev. Cell *4*, 67–82.

36. Li, S., Wang, L., Berman, M., Kong, Y.Y., and Dorf, M.E. (2011). Mapping a dynamic innate immunity protein interaction network regulating type I interferon production. Immunity *35*, 426–440.

37. Čajánek, L., Glatter, T., and Nigg, E.A. (2015). The E3 ubiquitin ligase Mib1 regulates Plk4 and centriole biogenesis. J. Cell Sci. *128*, 1674–1682.

38. Komander, D., and Rape, M. (2012). The ubiquitin code. Annu. Rev. Biochem. *81*, 203–229.

39. Lamb, C.A., Yoshimori, T., and Tooze, S.A. (2013). The autophagosome: origins unknown, biogenesis complex. Nat. Rev. Mol. Cell Biol. *14*, 759–774.

40. Füllgrabe, J., Klionsky, D.J., and Joseph, B. (2014). The return of the nucleus: transcriptional and epigenetic control of autophagy. Nat. Rev. Mol. Cell Biol. *15*, 65–74.

41. Pampliega, O., Orhon, I., Patel, B., Sridhar, S., Díaz-Carretero, A., Beau, I., Codogno, P., Satir, B.H., Satir, P., and Cuervo, A.M. (2013). Functional interaction between autophagy and ciliogenesis. Nature *502*, 194–200.

42. Mackeh, R., Perdiz, D., Lorin, S., Codogno, P., and Poüs, C. (2013). Autophagy and microtubules - new story, old players. J. Cell Sci. *126*, 1071–1080.

43. Pampliega, O., and Cuervo, A.M. (2016). Autophagy and primary cilia: dual interplay. Curr. Opin. Cell Biol. *39*, 1–7.

44. Wang, G., Chen, Q., Zhang, X., Zhang, B., Zhuo, X., Liu, J., Jiang, Q., and Zhang, C. (2013). PCM1 recruits Plk1 to the pericentriolar matrix to promote primary cilia disassembly before mitotic entry. J. Cell Sci. *126*, 1355–1365.

45. Szalai, P., Hagen, L.K., Sætre, F., Luhr, M., Sponheim, M., Øverbye, A., Mills, I.G., Seglen, P.O., and Engedal, N. (2015). Autophagic bulk sequestration of cytosolic cargo is independent of LC3, but requires GABARAPs. Exp. Cell Res. *333*, 21–38.

46. Chen, Z.W., Chang, C.S., Leil, T.A., and Olsen, R.W. (2007). C-terminal modification is required for GABARAP-mediated GABA(A) receptor trafficking. J. Neurosci. *27*, 6655–6663.

47. Skytte Rasmussen, M., Mouilleron, S., Kumar Shrestha, B., Wirth, M., Lee, R., Bowitz Larsen, K., Abudu Princely, Y., O'Reilly, N., Sjøttem, E., Tooze, S.A., et al. (2017). ATG4B contains a C-terminal LIR motif important for binding and efficient cleavage of mammalian orthologs of yeast Atg8. Autophagy *13*, 834–853.

48. Hornbeck, P.V., Zhang, B., Murray, B., Kornhauser, J.M., Latham, V., and Skrzypek, E. (2015). PhosphoSitePlus, 2014: mutations, PTMs and recalibrations. Nucleic Acids Res. *43*, D512–D520.

49. Subramani, S., and Malhotra, V. (2013). Non-autophagic roles of autophagy-related proteins. EMBO Rep. *14*, 143–151.

50. Polson, H.E.J., de Lartigue, J., Rigden, D.J., Reedijk, M., Urbé, S., Clague, M.J., and Tooze, S.A. (2010). Mammalian Atg18 (WIPI2) localizes to omegasome-anchored phagophores and positively regulates LC3 lipidation. Autophagy *6*, 506–522.

51. Webber, J.L., and Tooze, S.A. (2010). Coordinated regulation of autophagy by p38alpha MAPK through mAtg9 and p38IP. EMBO J. *29*, 27–40.

52. Orchard, S., Ammari, M., Aranda, B., Breuza, L., Briganti, L., Broackes-Carter, F., Campbell, N.H., Chavali, G., Chen, C., del-Toro, N., et al. (2014). The MIntAct project—IntAct as a common curation platform for 11 molecular interaction databases. Nucleic Acids Res. *42*, 358–363.

53. Cox, J., Matic, I., Hilger, M., Nagaraj, N., Selbach, M., Olsen, J.V., and Mann, M. (2009). A practical guide to the MaxQuant computational platform for SILAC-based quantitative proteomics. Nat. Protoc. *4*, 698–705.

54. MacLean, B., Tomazela, D.M., Shulman, N., Chambers, M., Finney, G.L., Frewen, B., Kern, R., Tabb, D.L., Liebler, D.C., and MacCoss, M.J. (2010). Skyline: an open source document editor for creating and analyzing targeted proteomics experiments. Bioinformatics *26*, 966–968.

55. Frank, R. (2002). The SPOT-synthesis technique. Synthetic peptide arrays on membrane supports–principles and applications. J. Immunol. Methods *267*, 13–26.

56. Axe, E.L., Walker, S.A., Manifava, M., Chandra, P., Roderick, H.L., Habermann, A., Griffiths, G., and Ktistakis, N.T. (2008). Autophagosome formation from membrane compartments enriched in phosphatidylinositol 3-phosphate and dynamically connected to the endoplasmic reticulum. J. Cell Biol. *182*, 685–701.

57. Lamb, C.A., Joachim, J., and Tooze, S.A. (2017). Quantifying autophagic structures in mammalian cells using confocal microscopy. Methods Enzymol. *587*, 21–42.

58. Razi, M., and Tooze, S.A. (2009). Correlative light and electron microscopy. Methods Enzymol. *452*, 261–275.

STAR★METHODS

KEY RESOURCES TABLE

REAGENT or RESOURCE	SOURCE	IDENTIFIER
**Antibodies**		
Mouse anti-Vinculin	Sigma-Aldrich	Cat#V9264; RRID: AB_10603627
Mouse anti-GABARAP, for IP	MBL International	Cat#M135-3; RRID: AB_10364779
Mouse anti-LC3, for IF (Clone 5F10)	NanoTools	Cat#0231-100/LC3-5F10
Mouse anti-GM130, for IF	BD Biosciences	Cat#610822; RRID: AB_398141
Mouse anti-PCM1, for WB	Atlas Antibodies	Cat#AMAb90565
Mouse anti-PCM1, for IF	Sigma-Aldrich	Cat#SAB1406228; RRID: AB_10738915
Mouse anti-Ubiquitin (Clone FK2)	MBL International	Cat#D058-3; RRID: AB_592937
Mouse anti-γ-tubulin ascites (Clone GTU-88)	Sigma-Aldrich	Cat#T6557; RRID: AB_477584
Mouse anti-p62/SQSTM1	BD Biosciences	Cat#610832; RRID: AB_398151
Mouse anti-p62/SQSTM1	Abnova Corporation	Cat#H00008878-M01; RRID: AB_437085
Mouse anti-FLAG (Clone M2)	Sigma-Aldrich	F3165
Mouse anti-WIPI2	[50]	N/A
Mouse anti-GFP (Clone 3E1)	Raised in-house, Cancer Research UK	N/A
Rabbit anti-Pericentrin	Abcam	Cat#ab4448; RRID: AB_304461
Rabbit anti-Mib1	Sigma-Aldrich	Cat#M5948; RRID: AB_1841007
Rabbit anti-Ubiquitin Lys48-specific (Clone APU2)	Millipore	Cat#05-1307; RRID: AB_1587578
Rabbit anti-ubiquitin Lys63-specific (Clone APU3)	Millipore	Cat#05-1308; RRID: AB_1587580
Rabbit anti-PCM1, for IF	Cell Signaling Technology	Cat#5213S; RRID: AB_10556960
Rabbit anti-ULK1 (H-240), for WB	Santa Cruz Biotechnology	Cat#sc-33182; RRID: AB_2214706
Rabbit anti-ULK1 (D8H5), for IF	Cell Signaling Technology	Cat#8054; RRID: AB_11178668
Rabbit anti-GABARAP	Abgent	Cat#AP1821a; RRID: AB_2278762
Rabbit anti-NBR1	Cell Signaling Technology	Cat#9891S; RRID: AB_10949888
Rabbit anti-HA	Covance Research Products Inc	Cat#PRB-101 also PRB-101P-500, PRB-101C-500, PRB-101C-200, PRB-101P-200; RRID: AB_291552
Rabbit anti-WIPI2	[50]	N/A
Rabbit anti-Actin	Abcam	Cat#ab8227; RRID: AB_2305186
Rabbit anti-LC3B	Abcam	Cat#ab48394; RRID: AB_881433
Hamster anti-ATG9	[51]	N/A
Guinea pig anti-p62	Progen Biotechnik	Cat#GP62-C
Goat anti-SSX2IP	Thermo Fisher Scientific	Cat#PA5-18258; RRID: AB_10985892
Goat anti-γ-tubulin (C-20)	Santa Cruz Biotechnology	Cat#sc-7396; RRID: AB_2211262
Mouse TrueBlot ULTRA: Anti-Mouse Ig HRP	Rockland Immunochemicals	Cat#18-8817-31; RRID: AB_2610850
Rabbit TrueBlot: Anti-Rabbit IgG HRP	Rockland Immunochemicals	Cat#18-8816-31; RRID: AB_2610847
Anti-GST HRP Conjugate	GE Healthcare	Cat#RPN1236; RRID: AB_771429
GFP-Trap_A	ChromoTek	Cat#gta-20; RRID: AB_2631357
Anti-HA Affinity Matrix (Clone 3F10)	Sigma-Aldrich	Cat#000000011815016001; RRID: AB_390914
**Bacterial and Virus Strains**		
E. coli BL21-CodonPlus(DE3)-RIL	Agilent	Cat#230245
**Chemicals, Peptides, and Recombinant Proteins**		
Lipofectamine 2000 transfection reagent	Thermo Fisher Scientific	Cat#11668019
Bafilomycin A1	Merck Millipore	Cat#196000
MG132	Merck Millipore	Cat#474790
Chloroacetamide	Sigma-Aldrich	Cat#C0267

(*Continued on next page*)

Continued

REAGENT or RESOURCE	SOURCE	IDENTIFIER
Nocodazole	Sigma-Aldrich	Cat#M1404
PhosSTOP	Sigma-Aldrich	Cat#000000004906845001
SignalBoostImmunoreaction Enhancer Kit	Merck Millipore	Cat#407207
Luminata Crescendo Western HRP substrate	Merck Millipore	Cat#WBLUR0100
Protein G Sepharose	Sigma-Aldrich	Cat#P3296-1ML
Glutathione Sepharose 4B	GE Healthcare	Cat#17075601
Dynabeads Protein A for Immunoprecipitation	Thermo Fisher Scientific	Cat#10001D
N-Ethylmaleimide	Sigma-Aldrich	Cat#E3876
Recombinant protein: GST	This paper	N/A
Recombinant protein: GST-GABARAP	This paper	N/A
cOmplete, EDTA-free Protease Inhibitor Cocktail	Sigma-Aldrich	Cat#000000005056489001
Critical Commercial Assays		
QuikChange Multi Site-Directed Mutagenesis Kit	Agilent	Cat#200515
Q5 Site-Directed Mutagenesis Kit	New England BioLabs	Cat#E0554S
Deposited Data		
The protein interactions from this publication have been submitted to the IMEx (http://www.imexconsortium.org) consortium through IntAct [52]	This paper	IM-25779
Experimental Models: Cell Lines		
Human: HEK293A cells	Thermo Fisher Scientific	Cat#R70507
Oligonucleotides		
siRNA targeting sequence RISC Free control	Dharmacon	Cat#D-001220-01
siRNA targeting sequence human GABARAP: GGUCAGUUCUACUUCUUGA	Dharmacon	Cat#D-012368-02
siRNA targeting sequence human PCM1: GCAAAUAGAUCAUCAGAAA	Dharmacon	Cat#D-005165-01
Primer: PCM1 SDM Primer 3xAla, CTG GTA ATA TAA GTC AAA AGT CTG ATG AAG AAG CTG CTG TAA AAG CTG AAG ATT TAC CAC TGA AAC TGA CAA TAT ATT C	This paper, see Method Details	N/A
Primer: PCM1 SDM Primer siRNA resistant Forward, CGT CGG AAA AGA ATA AGA AAA AGT TTG GTG TAG	This paper, see Method Details	N/A
Primer: PCM1 SDM Primer siRNA resistant Reverse, ACC TGT TCG CTT TCT TCT GTT GGG CAC C	This paper, see Method Details	N/A
Recombinant DNA		
pLVX-IRES-PURO 3xFLAG	[17]	N/A
pLVX-IRES-PURO 3xFLAG-Mib1	[17]	N/A
pLVX-IRES-PURO 3xFLAG-Mib1 C985S	[17]	N/A
pcDNA3 FLAG-Mib1	[34]	N/A
pcDNA3 FLAG-Mib1 C997S	[34]	N/A
pcDNA3 FLAG-Mib1 aa1-729	[34]	N/A
pcDNA3 FLAG-Mib1 aa730-1007	[34]	N/A
pcDNA3 FLAG-Mib1 aa1-429	[34]	N/A
pcDNA3 FLAG-Mib1 aa430-1007	[34]	N/A
pcDNA3 FLAG-Mib1 aa430-729	[34]	N/A
pcDNA3 FLAG-Mib1 aa820-1007	[34]	N/A
pEGFP C2 PCM1	[33]	N/A
pEGFP C2 PCM1 S372A	[33]	N/A
pEGFP C2 PCM1 S372E	[33]	N/A
pDEST EGFP-LC3A	Terje Johansen	N/A
pDEST EGFP-LC3B	Terje Johansen	N/A

(*Continued on next page*)

Continued		
REAGENT or RESOURCE	SOURCE	IDENTIFIER
pDEST EGFP-LC3C	Terje Johansen	N/A
pDEST EGFP-GABARAP	Terje Johansen	N/A
pDEST EGFP-GABARAPL1	Terje Johansen	N/A
pDEST EGFP-GABARAPL2	Terje Johansen	N/A
pDEST-myc-GABARAP	Terje Johansen	N/A
pDEST-EGFP-GABARAP G116A	[13]	N/A
pGEX-5X-1 GST-GABARAP	Zvulun Elazar	N/A
Software and Algorithms		
ImageJ	NIH	https://imagej.nih.gov/ij/; RRID: SCR_003070
MaxQuant	[53]	Version 1.3.0.5; RRID: SCR_014485
Skyline	[54]	Version 3.6.0.10162; RRID: SCR_014080
ZEN software	Zeiss	https://www.zeiss.com/
Imaris	Bitplane	Version 8; RRID: SCR_007370
iLIR	[23]	N/A
GraphPad Prism	GraphPad Software	Version 6; RRID: SCR_002798
Other		
Zeiss LSM 710 confocal microscope	Zeiss; The Francis Crick Institute	https://www.zeiss.com/
MultiPep automated peptide synthesizer	INTAVIS Bioanalytical Instruments AG, Germany [55]; The Francis Crick Institute	N/A
UltiMate 3000 RSLCnano System	Thermo Fisher Scientific; The Francis Crick Institute	Cat#ULTIM3000RSLCNANO
50-cm EasySpray PepMap column	Thermo Fisher Scientific; The Francis Crick Institute	N/A
EasySpray nano source	Thermo Fisher Scientific; The Francis Crick Institute	N/A
Q Exactive mass spectrometer	Thermo Fisher Scientific; The Francis Crick Institute	N/A

CONTACT FOR REAGENT AND RESOURCE SHARING

Further information and requests for resources and reagents should be directed to and will be fulfilled by the Lead Contact, Sharon A. Tooze (sharon.tooze@crick.ac.uk).

EXPERIMENTAL MODEL AND SUBJECT DETAILS

Cell lines and culture

HEK293A, U2OS, RPE-1, MEF GFP-DFCP1, HEK293 GFP-WIPI2b, HEK293 GFP-DFCP1 and their derivatives were grown in a humidified incubator at 37°C in 10% CO_2 in full medium: DMEM supplemented with 10% (20% for RPE-1 cells) fetal calf serum and 4 mM L-glutamine. To induce autophagy, cells were washed 3 times with Earle's balanced salt solution (EBSS) and incubated in EBSS for two hours. Where indicated, cells were treated with: 100 μg/mL Cycloheximide, 100 nM Bafilomycin A1 (Calbiochem), 10 μM MG132 (Calbiochem) or 50 μM Nocodazole (Sigma) for the specified time. GFP-DFCP1 MEF cells were a kind gift from Noboru Mizushima, (University of Tokoyo, Japan). The HEK293 GFP-WIPI2b stably expressing cells were as described [3]. RPE-1 control and CRISPR/Cas9 PCM1 knockout cells were as described [17]. HEK293 Flp-In T-Rex GFP-GABARAP cells were maintained in full medium + 200 μg/ml Hygromycin B + 5 μg/ml Blasticidin and induced for 24 hr with 1 μg/ml tetracycline in full medium to express GFP-GABARAP. HEK293 Flp-In T-Rex GFP-GABARAP cells were a kind gift from Anne Simonsen, (University of Oslo, Norway). The HEK293 GFP-DFCP1 stably expressing cells were a gift from N. Ktistakis (clone 201) [56] and maintained in the presence of G418 at μg/ml.

Organisms for recombinant protein expression

Escherichia coli cells were cultured in LB medium (see Method Details).

METHOD DETAILS

siRNA/DNA transfection and antibodies

Lipofectamine 2000 (Life Technologies) was used for transient transfection of cells according to the manufacturer's instructions. DNA plasmids were used at a concentration of 1 μg/mL of transfection mix. Where indicated 3xFLAG pLVX-IRES-PURO was used as a vector control. For RNAi, cells were transfected with the relevant siRNA oligo using Lipofectamine 2000 (Life Technologies). Cells were harvested 72 hr after transfection. Final concentration of siRNA oligos was 37.5 nM. siRNA oligos used (Dharmacon): D-001220-01 (RISC-Free, control), D-012368-02 (GABARAP) and D-005165-01 (PCM1).

EGFP-PCM1 (pEGFP C2) 3xAla D1954A, F1955A, V1958A point mutations were generated by using QuikChange Multi Site-Directed Mutagenesis Kit (Agilent Technologies). EGFP-PCM1 wild-type and 3xAla constructs resistant to PCM1 siRNA D-005165-01 (Dharmacon) were generated using Q5 Site-Directed Mutagenesis Kit (NEB, E0554S). 3xFLAG pLVX-IRES-PURO, 3xFLAG-Mib1 pLVX-IRES-PURO and 3xFLAG-Mib1 C985S pLVX-IRES-PURO were as described [17]. FLAG-Mib1 pCDNA 3 truncations aa1-729, aa730-1007, aa1-429, aa430-1007, aa430-1007, aa430-729, aa820-1007 and C997S mutant were a gift from Jason Berndt (Howard Hughes Medical Institute, USA) and as described [34]. EGFP-PCM1 (NP_001302436) (pEGFP C2) and S372A/E were gifts from Takashi Toda (Hiroshima University, Japan) [33]. pDEST EGFP-mAtg8 homologs and pDEST-myc-GABARAP (human) were a gift from Terje Johansen (UiT, The Arctic University of Norway, Tromsø). pDEST-EGFP-GABARAP G116A mutant was generated by us previously [13].

Mouse antibodies: anti-Vinculin (Sigma, V9264), anti-GABARAP (MBL, M135-3) for immunoprecipitation, anti-LC3 for IF (5F10) (Nanotools, 0231-100/LC3-5F10), anti-GM130 (for IF) (BD Biosciences, 610822), anti-PCM1 (for WB Atlas antibodies, AMAb90565; for IF Sigma, SAB1406228), anti-ubiquitin (FK2) (MBL, D058-3), anti-γ-tubulin ascites (Sigma, GTU-88, T6557), anti-p62 (BD Biosciences, 610832; Abnova, H00008878-M01), anti-FLAG M2 (Sigma), anti-GFP (CRUK, 3E1), anti-WIPI2 [50]. Rabbit antibodies: anti-Pericentrin (Abcam, ab4448), anti-Mib1 (Sigma, M5948), anti-Ubiquitin Lys48 linked (APU2) (Millipore, 05-1307), anti-Ubiquitin Lys63 linked (APU3) (Millipore, 05-1308), anti-PCM1 (for IF, Cell Signaling, 5213), anti-ULK1 (for WB, Santa Cruz, sc-33182; for IF, Cell Signaling, 8054 D8H5), anti-GABARAP (Abgent, AP1821a), anti-NBR1 (D2E6) (Cell Signaling, 9891), anti-HA (Covance, PRB-101C), anti-WIPI2 [50], anti-Actin (Abcam, ab8227), anti-LC3 for WB (Abcam, ab48394). Hamster antibodies: anti-Atg9 [51]. Guinea pig antibodies: anti-p62 (for IF) (Progen, GP62-C). Goat antibodies: anti-SSX2IP (ThermoFisher, PA5-18258), anti-γ-tubulin (C-20) (Santa Cruz, sc-7396). Antibodies were used at manufacturer's suggested concentrations. Secondary antibodies for IF, from Life Technologies unless otherwise specified, were anti-rabbit IgG Alexa Fluor 488, 555 and 647, anti-mouse IgG Alexa Fluor 488, 647 and 350, anti-goat IgG Alexa Fluor 647, anti-guinea pig Alexa Fluor 555 and anti-hamster Cy3 (Jackson ImmunoResearch). HRP-conjugated secondary antibodies used for WB were from GE Healthcare.

Western Blotting

Cells were lysed in ice-cold TNTE buffer (20 mM Tris-HCl, pH 7.4, 150 mM NaCl, 0.5% w/v Triton X-100, 5 mM EDTA) containing EDTA-free Complete Protease Inhibitor cocktail (Roche) and PhosSTOP (Roche). Lysates were cleared by centrifugation and resolved on NuPAGE Bis-Tris 4%–12% gels (Life Technologies) (or 4%–20% Tris-Glycine gels for GABARAP lipidation assays) followed by transfer onto a PVDF membrane (Millipore). Following incubation with primary and secondary antibodies the blots were developed by enhanced chemiluminescence (GE Healthcare). Densitometry was performed with ImageJ software. For western blotting of weak signal antibodies, primary antibody was diluted with SignalBoost Immunoreaction Enhancer Kit (Merck Millipore, 407207) and blots were developed with Luminata Crescendo Western HRP substrate (Merck Millipore).

Immunoprecipitation

Cells were lysed using TNTE buffer (20 mM Tris-HCl pH 7.4, 150 mM NaCl, 5 mM EDTA, 0.5% Triton X-100, 1x Complete protease inhibitor (Roche), 1x PhosSTOP (Roche)) supplemented with 10% (v/v) glycerol and 0.1% (w/v) BSA and the clarified lysates used for immunoprecipitation using the indicated antibodies for 2 hr at 4°C. Antibodies were coupled to protein G Sepharose (Sigma). Pelleted beads were washed 3 times with TNTE buffer and eluted with 2x Laemmli sample buffer at 100°C for 10 min before resolving by SDS-PAGE (4%–12% Bis-Tris NuPAGE gels, Life Technologies) and western blotting. GFP-tagged proteins were immunoprecipitated using GFP-TRAP beads and HA-tagged proteins with anti-HA affinity matrix 3F10 (Roche), using the same buffer and protocol. During western blotting of IP experiments, TrueBlot (Rockland) was used to reduce background from IgG when required.

Immunoisolation of GFP-DFCP1 membranes

HEK293 cells stably expressing GFP-DFCP1 were treated with EBSS for 2 hr. Cells were then washed in ice cold PBS and harvested by centrifugation at 200 x g at 4°C. Pellets were resuspended using an ice cold buffer (20mM HEPES, pH 7.4; 250mM sucrose; 1mM EDTA) supplemented with EDTA-free Complete protease inhibitor cocktail (Roche). The resuspended pellet was then passed through a 27G needle for homogenization before clarification by centrifugation at 3000 x g at 4°C. Supernatants were used for incubation overnight at 4°C with mouse anti-GFP antibody protein A Dynabeads. The GFP-DFCP1-positive membranes on the beads were then washed 3 times (20mM HEPES, pH 7.4; 250mM sucrose; 1mM EDTA:75mM NaCl) and eluted with 2x laemmli sample buffer before resolving by SDS-PAGE and western blotting.

GST pulldowns

GST or GST-GABARAP was expressed in *E. coli* BL21-CodonPlus(DE3)-RIL (Agilent) cells in LB medium. Human GST-GABARAP pGEX-5X-1 was a gift from Zvulun Elazar (Weizmann Institute of Science, Israel). Expression was induced by addition of 1 mM IPTG at OD_{600} = 0.6 and cells were incubated at 37°C for 4 hr. Harvested cells were lysed using sonication on ice in a lysis buffer (PBSA + 1% Triton X-100, supplemented with 1 x Complete protease inhibitor (Roche)) and the clarified supernatant was subsequently applied to Glutathione Sepharose 4B beads (GE Healthcare). After several washes with (PBSA + 1% Triton X-100 + 500 mM NaCl supplemented with 1 x Complete protease inhibitor (Roche)), fusion protein-bound beads were used directly in GST pulldown assays. For GST pulldowns, HEK293A lysate was incubated with immobilized GST or GST-GABARAP on glutathione beads for 2 hr at 4°C in TNTE buffer supplemented with 10% (v/v) glycerol and 0.1% (w/v) BSA. Beads were then washed 3 x with TNTE before SDS-PAGE and western blotting.

Peptide Arrays and GST Overlay Assay

SPOT synthesis of peptide arrays on cellulose membranes were performed using a MultiPep automated peptide synthesizer (INTAVIS Bioanalytical Instruments AG, Germany) as previously described [55]. After blocking the cellulose membranes in TBST with 5% nonfat dry milk, peptide interactions with GST or GST fusion proteins were tested by overlaying the membranes with 1 μg/ml of recombinant protein for 2 hr at room temperature. Filters were washed in TBST, and bound proteins were detected with HRP-conjugated anti-GST antibody (1:5000; clone RPN1236; GE Healthcare).

Ubiquitination assays

Where indicated cells were treated with 10 μM MG132 for 5 hr prior to lysis. To inhibit deubiquitinases cells were lysed in TNTE buffer (20 mM Tris-HCl pH 7.4, 150 mM NaCl, 5 mM EDTA, 0.5% Triton X-100, 1x Complete protease inhibitor (Roche), 1x PhosSTOP (Roche)) supplemented with 20 mM N-Ethylmaleimide (NEM) prior to immunoprecipitation as described. Where indicated GFP-TRAP immunoprecipitates were washed 3 x in denaturing buffer (8 M Urea, 1% SDS in PBS) at room temperature before SDS-PAGE to remove binding partners.

Alternatively cells were lysed in boiling SDS buffer (2% SDS, 1 mM EDTA, 50 mM NaF, preheated at 110°C) and then diluted with 4 volumes of dilution buffer (2.5% Triton X-100, 12.5 mM Tris pH 7.5, 187.5 mM NaCl and 1x Complete protease inhibitor (Roche), 1x PhosSTOP (Roche)) to a final concentration of 0.4% SDS, 2% TX100. Diluted lysates were clarified by centrifugation and subjected to anti-HA immunoprecipitation overnight. Immunoprecipitates were washed 3x with wash buffer (1 volume SDS buffer, 4 volumes dilution buffer) before SDS-PAGE.

Ubiquitination mass spectrometry

Cells were lysed in ice-cold buffer (Tris-HCl 20 mM pH6.8, 0.5% (w/v) Triton X-100, 150 mM NaCl, 5 mM EDTA, phosphatase inhibitor cocktail and mammalian protease inhibitor cocktail [Roche], and 20 mM N-Ethylmaleimide). The lysates were then clarified by centrifugation (5 min, full speed, 4°C) and the supernatants containing GFP-GABARAP ubiquitinated conjugates were subjected to immunoprecipitation at 4°C with GFP-TRAP beads for 2 hr. Immunoprecipitates were washed three times with stringent denaturing washing buffer (8 M Urea, 1% SDS in PBS) at room temperature to remove GABARAP binding partners and then once with 10 mM Tris pH 7.5 before preparation for SDS-PAGE. Proteins were resolved by SDS-PAGE, fixed, and stained with GelCode, and gel slices subjected to tryptic digestion and mass spectrometry analysis (below).

Three bands covering an entire SDS-PAGE lane were excised for each sample. The excised gel pieces were de-stained with 50% acetonitrile, 100 mM ammonium bicarbonate, reduced with 10 mM DTT and alkylated with 20 mM chloroacetamide (all reagents from Sigma-Aldrich). After alkylation, the proteins were digested with 350 ng trypsin overnight at 37°C. The resulting peptides were extracted in 0.1% TFA and speed vacuum dried. For MS analysis, peptides were re-suspended in 0.1% TFA and loaded onto 50-cm Easy Spray PepMap column (Thermo Fisher Scientific). Reverse phase chromatography was performed using the RSLC nano U3000 (Thermo Fisher Scientific) with a binary buffer system at a flow rate of 250 nL/min. The in-gel digested samples were run on a linear gradient of solvent B (2- 40%) in 34 min, total run time of 60 min including column conditioning. The nanoLC was coupled to a Q Exactive mass spectrometer using an EasySpray nano source (Thermo Fisher Scientific). The Q Exactive was operated in data-dependent acquisition mode acquiring HCD MS/MS scans (R = 17,500) after an MS1 scan (R = 70, 000) on the 10 most abundant ions using MS1 target of 1×10^6 ions, and MS2 target of 5×10^4 ions. The maximum ion injection time utilized for MS2 scans was 120 ms, the HCD normalized collision energy was set at 28, the dynamic exclusion was set at 10 s, and the peptide match and isotope exclusion functions were enabled.

For the PRM (Parallel Reaction Monitoring) experiments, the QExactive was operated in data independent mode. A full scan MS1 was measured at 70,000 resolution (AGC target 1×10^6, 200 ms maximum injection time, m/z 300−1200) followed by seven PRM scans at 17,500 resolution as triggered by an inclusion list. (AGC target 2×10^5, 50 ms maximum injection time). Ion activation/dissociation was performed using HCD at normalized collision energy of 28.

For identification of diGly containing peptides, raw data files were analyzed with MaxQuant software (version 1.3.0.5) as described previously [53]. Parent ion and tandem mass spectra were searched against UniprotKB *Homo sapiens* database. A list of 247 common laboratory contaminants provided by MaxQuant was also added to the database. For the search the enzyme specificity was set

to trypsin with maximum of three missed cleavages. The precursor mass tolerance was set to 20 ppm for the first search (used for mass re-calibration) and to 6 ppm for the main search. Carbamidomethylation of cysteines was specified as fixed modification, oxidized methionines, N-terminal protein acetylation and di-glycine-lysine were searched as variable modifications. The datasets were filtered on posterior error probability to achieve 1% false discovery rate on protein, peptide and site level.

The PRM data was analyzed using Skyline 3.6.0.10162 software [54]. The spectral library was built in Skyline using the MaxQuant msms.txt file and the BiblioSpec algorithm. The background proteome was generated using UniprotKB *Homo sapiens* database. Precursor and product ion extracted chromatograms (XICs) were generated using the following settings in Skyline. Signal extraction was performed on +2, +3, +4 precursor ions and +1 and +2 b and y fragment ions. Retention time filtering was restricted during import to be within 5 min of MS/MS ID times and ion mass tolerance was set to 0.055 m/z. A peptide was considered identified if at least five overlapping transitions were detected. Quantitation was performed using MS1 XICs where three replicate measurements were performed. To confirm that the changes in abundance of the diGly containing peptides are not a result of changes in the overall abundance of the GABARAP protein, four unmodified peptides were also quantified (normalization peptides, Figure S5K).

Two GABARAP ubiquitination sites, lysine 13 (K13) and lysine 23 (K23), were identified by mass spectrometry on three different peptides (Figure 7K). Peptides containing these sites were quantified using integration of precursor ion signals with Skyline software. All measurements were done in triplicates and the mean and standard error of the peak area is displayed. To confirm that the total amount of the GABARAP protein is constant, four normalization peptides (unmodified) were also quantified (Figure S5K).

Confocal microscopy

Cells were grown on coverslips, fixed with 3% paraformaldehyde in PBS for 20 min before permeabilization with either, 0.2% Triton X-100 or 50 ug/mL digitonin in PBS for 3 min (ULK1 or ATG9 staining, respectively), or room temperature methanol for 5 min (all other antibodies). Coverslips were then blocked in 5% BSA in PBS (Roche) in PBS for 20 min. Coverslips were incubated with primary antibody in 1% BSA in PBS 1 hr at room temperature or 4°C overnight (ULK1 only). Coverslips were washed and incubated with secondary antibody in the same buffer as primary for 1 hr, before final washing with PBS and water. Images were acquired using a Zeiss LSM 710 confocal microscope and ZEN imaging software.

Confocal data quantification

Puncta formation and centrosomal intensity was quantified by Imaris image analysis software. For autophagosomal puncta formation, the Imaris ‘Spots’ function was used to segment puncta. Cells were counted manually. Spots per cell numbers were derived for whole fields of cells with 10 fields per condition, per experiment captured. Typically > 100 cells per condition per experiment were analyzed. A detailed protocol for spot counting autophagosomes using Imaris has previously been published [57]. Centrosomal intensity quantification of GABARAP and γ-tubulin was performed similarly using Imaris software. The ‘Surfaces’ function of Imaris was used to create a mask on the centrosomes (γ-tubulin signal) and the amount of GABARAP and γ-tubulin signal at this mask was quantified for individual centrosomes, as shown in Figure 4B. The arbitrary signal intensity values were normalized to the mean of the RISC Free values to create a normalized RISC Free mean of 1.

Pearson’s correlation coefficient was calculated per cell using the colocalization algorithm in Imaris. Non-centrosomal intracellular regions were chosen for analysis. Intracellular regions did not encompass the whole cytoplasmic area but rather a portion of a cell’s cytoplasm. Images were quantified from 2 experiments, in total: RISC Free 15 images, siPCM1 9 images and GABARAP-p62 16 images.

Correlative Light and Electron Microscopy

Correlative light and electron microscopy (CLEM) was performed as previously described [58]. HEK293A cells were fixed with PFA 2% + Glutaraldehyde 2% in PBS, followed by 0.2% Triton X-100 in PBS permeabilization and 2 × 5 min washes in 10% BSA in PBS. Cells were stained with the indicated antibodies as described above.

Primers used in this study

Primer	Description	Sequence (5′-3′)
PCM1 SDM Primer 3xAla	Mutation of hPCM1 (NP_001302436) LIR motif aa1953-EDFVKV-aa1958 to EAAVKA	CTG GTA ATA TAA GTC AAA AGT CTG ATG AAG AAG CTG CTG TAA AAG CTG AAG ATT TAC CAC TGA AAC TGA CAA TAT ATT C
PCM1 SDM Primer siRNA resistant Forward	Silent mutations of hPCM1 (NP_001302436) to make resistant to Dharmacon siRNA D-005165-01	cgt cgg aaa AGA ATA AGA AAA AGT TTG GTG TAG
PCM1 SDM Primer siRNA resistant Reverse	Silent mutations of hPCM1 (NP_001302436) to make resistant to Dharmacon siRNA D-005165-01	acc tgt tcg cTT TCT TCT GTT GGG CAC C

QUANTIFICATION AND STATISTICAL ANALYSIS

The statistical details of all experiments are reported in the figure legends and figures, including statistical analysis performed, error bars, statistical significance and exact n numbers. Statistics were performed using GraphPad Prism 6 software, as detailed in the figure legends. For further details of confocal data analysis and mass spectrometry data analysis and software used see Method Details.

DATA AND SOFTWARE AVAILABILITY

The protein interactions from this publication have been submitted to the IMEx (http://www.imexconsortium.org) consortium through IntAct [52] and assigned the identifier IM-25779.

Current Biology, Volume *27*

Supplemental Information

Centriolar Satellites Control GABARAP Ubiquitination and GABARAP-Mediated Autophagy

Justin Joachim, Minoo Razi, Delphine Judith, Martina Wirth, Emily Calamita, Vesela Encheva, Brian D. Dynlacht, Ambrosius P. Snijders, Nicola O'Reilly, Harold B.J. Jefferies, and Sharon A. Tooze

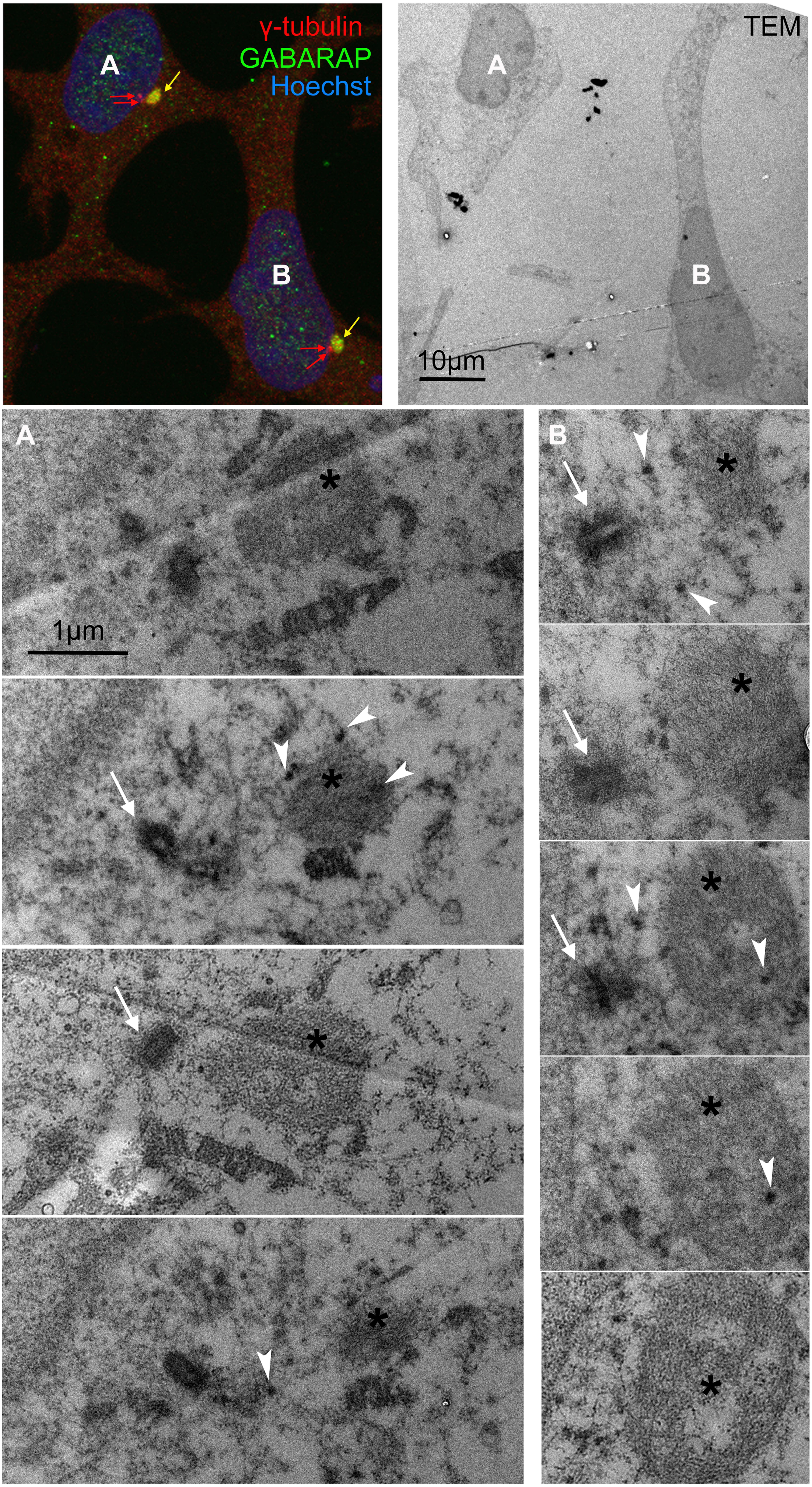
γ-tubulin
GABARAP
Hoechst
A
B
TEM
10µm
1µm

Figure S1. Correlative light and electron microscopy of centrosomal GABARAP. See also Figure 2.

HEK293A cells (**A** and **B**) fixed and Triton X-100 permeabilized were labelled with the indicated antibodies for confocal microscopy followed by transmission electron microscopy (TEM). Red arrows, γ-tubulin positive GABARAP negative centrioles. Yellow arrows, γ-tubulin positive and GABARAP positive pericentriolar material. TEM sections from the same cell (A or B) are shown. *, pericentriolar material; white arrows, centrioles; white arrowheads, detergent-resistant electron-dense granules, likely centriolar satellites.

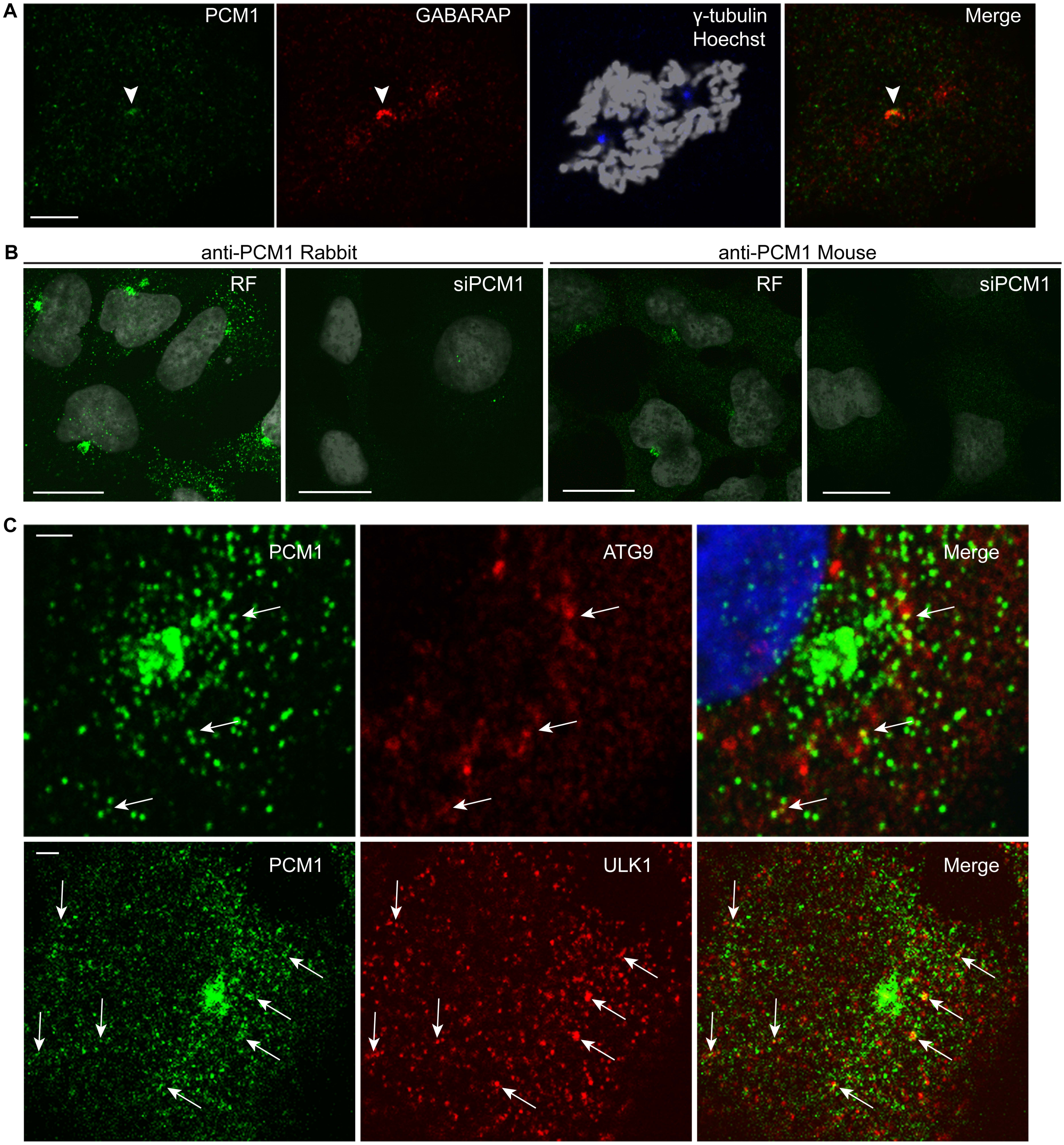
A
PCM1
GABARAP
γ-tubulin
Hoechst
Merge
B
anti-PCM1 Rabbit
anti-PCM1 Mouse
RF
siPCM1
RF
siPCM1
C
PCM1
ATG9
Merge
PCM1
ULK1
Merge

Figure S2. Validation of anti-PCM1 staining and PCM1 colocalization with autophagy markers. See also Figures 2 and 3.

A HEK293A cells incubated in full medium were fixed and labelled with the indicated antibodies. Scale bar, 5 µm. Mitotic cell is shown. Mouse anti-PCM1, rabbit anti-GABARAP and goat anti-γ tubulin antibodies were used. **B** HEK293A cells were treated with RF or PCM1 siRNA for 72 hr then, fixed, and labelled with the indicated PCM1 antibodies. Scale bar, 20 µm. **C** HEK293A cells starved for 2 hr in EBSS (ULK1 staining only) then fixed and labelled with the indicated antibodies. Scale bars, 2 µm. ATG9 labelled cell was incubated in full medium. Arrows indicate colocalization between PCM1 and autophagy markers. Hoechst DNA staining is shown in blue in the merge (ATG9 only). Rabbit anti-PCM1 was used with hamster anti-ATG9 antibody; mouse anti-PCM1 was used with rabbit anti-ULK1 antibody.

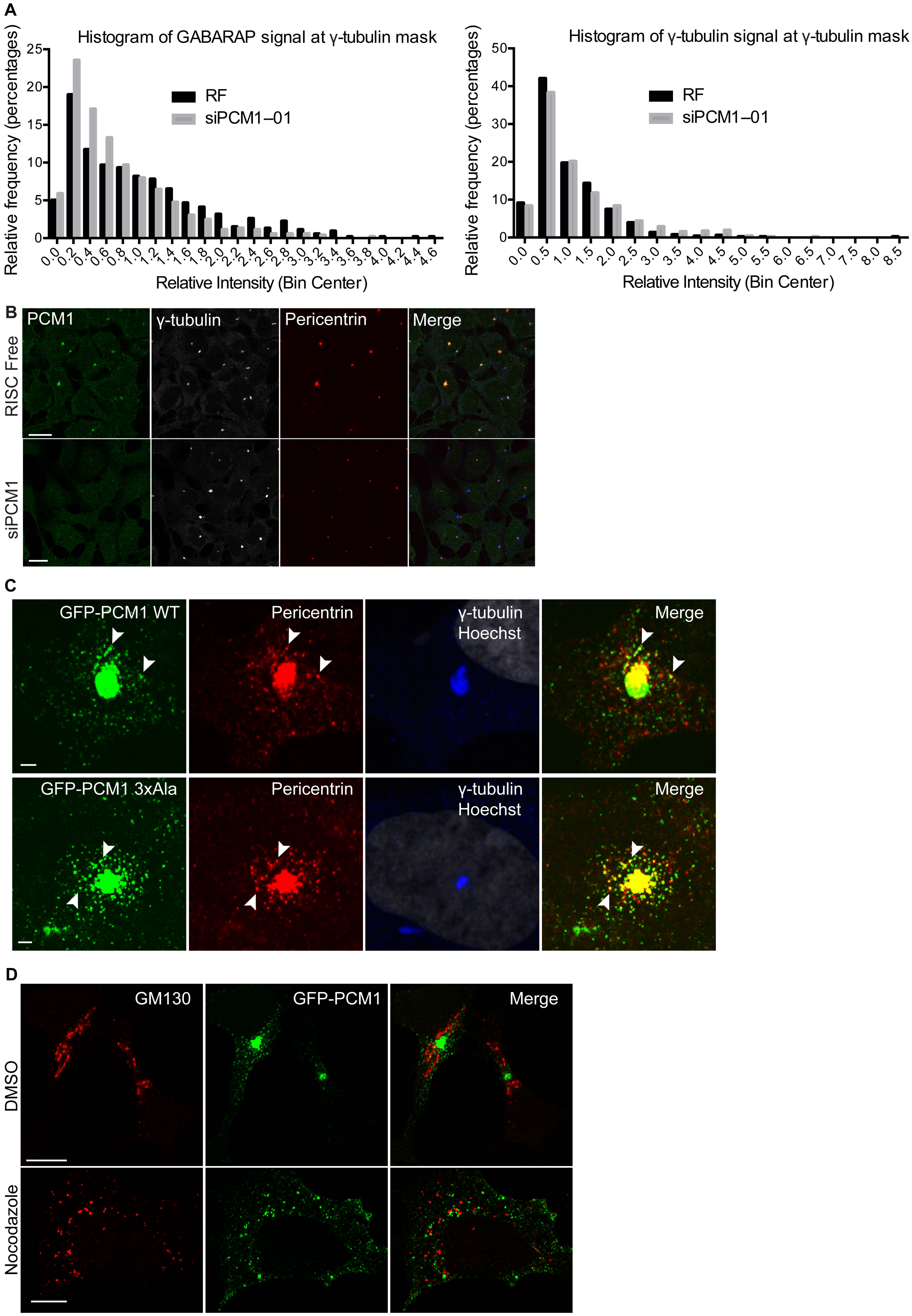
A
Histogram of GABARAP signal at γ-tubulin mask
Relative frequency (percentages)
Relative Intensity (Bin Center)
RF
siPCM1–01
Histogram of γ-tubulin signal at γ-tubulin mask
Relative frequency (percentages)
Relative Intensity (Bin Center)
RF
siPCM1–01
B
PCM1
γ-tubulin
Pericentrin
Merge
RISC Free
siPCM1
C
GFP-PCM1 WT
Pericentrin
γ-tubulin
Hoechst
Merge
GFP-PCM1 3xAla
Pericentrin
γ-tubulin
Hoechst
Merge
D
GM130
GFP-PCM1
Merge
DMSO
Nocodazole

Figure S3. PCM1 depletion affects centrosomal GABARAP and Pericentrin localization and the PCM1 LIR does not regulate colocalization with Pericentrin. See also Figure 4.

A Frequency distribution of data shown in Figure 4B. Data is normalized to the mean intensity of RF signals and assigned to the nearest bin center. **B** HEK293A cells were treated with RF or PCM1 siRNA for 72 hr then, fixed, and labelled with the indicated antibodies. Scale bar, 20 µm. Mouse anti-PCM1, goat anti-γ tubulin, and rabbit anti-Pericentrin antibodies were used. **C** HEK293A cells expressing GFP-PCM1 wild-type or 3xAla LIR mutant starved for 2 hr in EBSS then fixed and labelled with rabbit anti-Pericentrin and mouse anti-γ tubulin. Scale bars, 2µm. Hoechst DNA staining is shown in the γ-tubulin channel and false-coloured white. Merge shows red and green channels. Arrowheads, GFP-PCM1 WT or 3xAla colocalization with Pericentrin puncta. **D** HEK293A cells expressing GFP-PCM1 were treated with 50 µM nocodazole for 5 hr in total prior to fixation and labelled with mouse anti-GM130 antibodies. Scale bar, 10 µm.

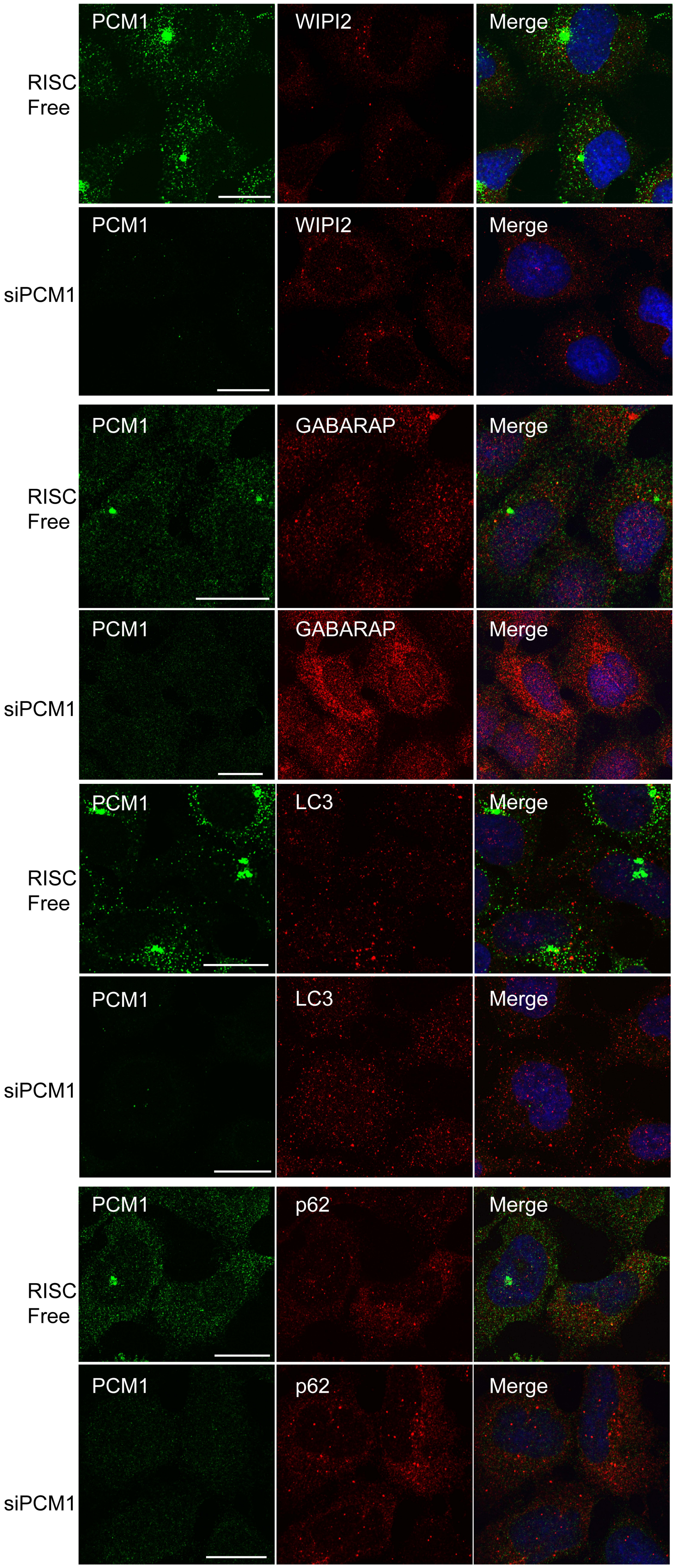
RISC Free
siPCM1
PCM1
WIPI2
GABARAP
LC3
p62
Merge

Figure S4. Depletion of PCM1 increases GABARAP but not LC3B specific autophagosome formation. See also Figure 4.

HEK293A cells were treated with RF or PCM1 siRNA for 72 hr and incubated in EBSS for 2 hr, fixed, and labelled with the indicated antibodies. Scale bars, 20 µm. Mouse anti-WIPI2, rabbit anti-PCM1, rabbit anti-GABARAP, mouse anti-LC3B, guinea pig anti-p62. For co-staining with rabbit anti-GABARAP, mouse anti-PCM1 was used.

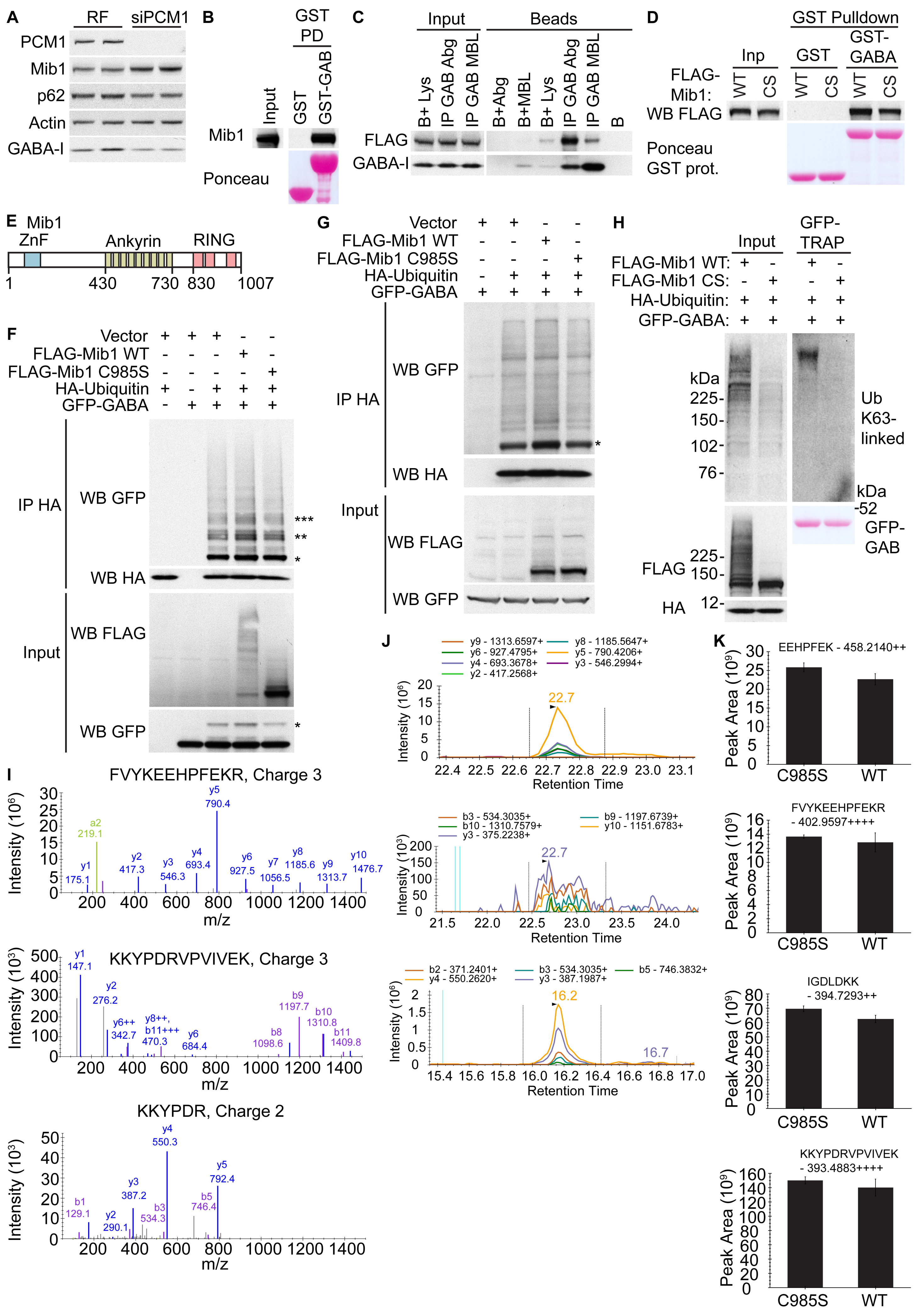

A
RF
siPCM1
PCM1
Mib1
p62
Actin
GABA-I
B
GST PD
Input
GST
GST-GAB
Mib1
Ponceau
C
Input
Beads
B+ Lys
IP GAB Abg
IP GAB MBL
B+Abg
B+MBL
B+ Lys
IP GAB Abg
IP GAB MBL
B
FLAG
GABA-I
D
GST Pulldown
Inp
GST
GST-GABA
FLAG-Mib1:
WB FLAG
WT
CS
Ponceau GST prot.
E
Mib1
ZnF
Ankyrin
RING
1
430
730
830
1007
F
Vector
FLAG-Mib1 WT
FLAG-Mib1 C985S
HA-Ubiquitin
GFP-GABA
IP HA
WB GFP
WB HA
Input
WB FLAG
WB GFP
***
**
*
G
Vector
FLAG-Mib1 WT
FLAG-Mib1 C985S
HA-Ubiquitin
GFP-GABA
IP HA
WB GFP
WB HA
Input
WB FLAG
WB GFP
H
Input
GFP-TRAP
FLAG-Mib1 WT:
FLAG-Mib1 CS:
HA-Ubiquitin:
GFP-GABA:
kDa
225-
150-
102-
76-
Ub K63-linked
kDa
-52
GFP-GAB
FLAG
225-
150-
HA
12-
I
FVYKEEHPFEKR, Charge 3
Intensity (10^6)
m/z
KKYPDRVPVIVEK, Charge 3
Intensity (10^3)
KKYPDR, Charge 2
J
y9 - 1313.6597+
y8 - 1185.5647+
y6 - 927.4795+
y5 - 790.4206+
y4 - 693.3678+
y3 - 546.2994+
y2 - 417.2568+
22.7
Intensity (10^6)
Retention Time
b3 - 534.3035+
b9 - 1197.6739+
b10 - 1310.7579+
y10 - 1151.6783+
y3 - 375.2238+
Intensity (10^3)
b2 - 371.2401+
b3 - 534.3035+
b5 - 746.3832+
y4 - 550.2620+
y3 - 387.1987+
16.2
16.7
K
EEHPFEK - 458.2140++
FVYKEEHPFEKR - 402.9597++++
IGDLDKK - 394.7293++
KKYPDRVPVIVEK - 393.4883++++
Peak Area (10^9)
C985S
WT

Figure S5. Mib1 promotes ubiquitination of GABARAP. See also Figure 7.
A HEK293A cells were treated with RF or PCM1 siRNA for 72 hr then immunoblotted. **B** HEK293A cells were incubated with recombinant GST or GST-GABARAP beads and immunoblotted. PD, pulldown. **C** Anti-GABARAP immunoprecipitate from HEK293A cells analyzed by immunoblotting. Abg, anti-GABARAP from Abgent. MBL, anti-GABARAP from MBL. Lys, HEK293A lysate. B, beads. **D** HEK293A cells expressing the indicated constructs were incubated with recombinant GST or GST-GABARAP beads and immunoblotted. Prot, protein; WT, wild-type; CS, C997S mutant. **E** Primary structure of human Mib1 showing domains. **F** Immunoprecipitation of U2OS cells lysed in boiling SDS buffer and expressing the indicated constructs and immunoblot. *, **, ***, mono, di and tri ubiquitinated GFP-GABARAP, respectively. WT, wild-type. **G** Immunoprecipitation of HEK293A cells lysed in TNTE buffer without N-Ethylmaleimide and expressing the indicated constructs and immunoblot. *, monoubiquitinated GFP-GABARAP. WT, wild-type. **H** GFP-TRAP of HEK293A cells expressing the indicated constructs and immunoblot. Immunoprecipitates were stringently washed in denaturing buffer. GAB, GABARAP; WT, wild-type; CS, C985S mutant. **I** Identification of GABARAP ubiquitination sites by LC MS/MS. GABARAP ubiquitination sites were identified by mass spectrometry following protein digestion with trypsin and LC MS/MS analysis using the QExactive MS. Two ubiquitination sites were detected including lysine 13 (K13) and lysine 23 (K23). The sites were identified with MaxQuant where the FDR was restricted to 1 % on peptide and site level. Top, MS2 fragmentation spectra of the FVYKEEHPFEK(diGly)R peptide containing K13. All matching b and y ions are annotated with their measured m/z (mass to charge). Middle and bottom, MS2 fragmentation spectra of peptides K(diGly)KYPDRVPVIVEK and K(diGly)KYPDR containing K23 ubiquitination site. The two peptides are a result of a missed cleavage and both contain the same ubiquitinated lysine. All matching y and b ions are annotated with their measured m/z (mass to charge). **J** Detection of GABARAP ubiquitination peptides using PRM. Three peptides of the GABARAP protein containing ubiquitinated lysines were detected using LC MS/MS. To further confirm their presence/absence a PRM-based targeted assay was developed using Skyline software. The observed MS2 signals (transitions) were integrated with Skyline and manually verified. Top, Co-eluting transitions of the K13 containing peptide FVYKEEHPFEK(diGly)R. The co-elution profiles of seven detectable y ions are annotated with their measured m/z (mass to charge) values. Middle and bottom, the K23 ubiquitination site was detected on two different peptides. Peptide K(diGly)KYPDRVPVIVEK (middle) and peptide K(diGly)KYPDR (bottom). For both peptides the co-elution profiles of five b and y ions are annotated with their measured m/z (mass to charge) values. **K** Quantitation of normalization peptides. To check for differences in the total amount of the GABARAP protein recovery, four unmodified peptides were also quantified. The peak areas of these normalization peptides were obtained using MS1 integration in Skyline. Each peak area was measured in three technical replicates. The mean peak area of the three measurements is displayed and the error bars indicate standard errors. Peak area calculations of the normalization peptides did not reveal any major differences in GABARAP loading between the different conditions. (Top) Peak areas of peptide EEHPFEK, (middle-top) peak areas of peptide FVYKEEHPFEKR, (middle-bottom) peak areas of peptide IGDLDKK, (bottom) peak areas of peptide KKYPDRVPVIVEK.
